# A spatiotemporally coordinated curcumin-based microneedle patch for SDF-1α delivery and synergistic myocardial infarction therapy

**DOI:** 10.1186/s12951-026-04473-4

**Published:** 2026-04-26

**Authors:** Xue-Yan Jiang, Yuan Luo, Yang Yang, Da-Wei Tang, Zhizhong Wang, Pei Huang, Fang-Zhen Wang, Shu-Meng Zhang, Hao-Min Zhang, Yi-Yun Ma, Xu-Chen Liu, Yun-Ru Li, Wenhua Zheng, Lingmin Zhang, Xi-Yong Yu, Gen He

**Affiliations:** 1 Key Laboratory of Molecular Target & Clinical Pharmacology, State Key Laboratory of Respiratory Disease, School of Pharmaceutical Sciences, the NMPA, Guangzhou Medical University, Guangzhou, 511436 China; 2 Department of Pharmaceutical Science, Faculty of Health Sciences, University of Macau, Taipa, 999078 Macau China

**Keywords:** Myocardial Infarction, Microneedle Patch, Curcumin, SDF-1α, Macrophage Reprogramming, Angiogenesis

## Abstract

**Introduction:**

Effective myocardial regeneration following infarction remains a major clinical challenge due to the complex and dynamic pathological microenvironment. Current clinical management fails to adequately modulate the dynamic infarct microenvironment, where dysregulated inflammation and insufficient angiogenesis represent key therapeutic targets.

**Methods:**

To address this challenge, we developed a spatiotemporally coordinated microneedle (MN) patch based on curcumin-conjugated gelatin methacrylate (Cur-GelMA) hydrogel for co-delivery of curcumin and stromal cell-derived factor-1α (SDF-1α).

**Results:**

The engineered Cur-GelMA network significantly enhanced curcumin solubility and bioavailability, while PDMS micromolding enabled fabrication of mechanically robust MN patches. This integrated system provides rapid, reactive oxygen species-responsive curcumin release along with sustained SDF-1α delivery, achieving spatially targeted penetration and localized drug deposition in the infarcted myocardium. In vitro studies demonstrated that curcumin-hydrogel effectively reprogrammed macrophage polarization from pro-inflammatory M1 to reparative M2 phenotype, downregulating pro-inflammatory cytokines while upregulating anti-inflammatory cytokine. Simultaneously, sustained SDF-1α release promoted endothelial cell proliferation, migration, and tube formation via VEGF pathway activation. In a rat MI model, the SDF-1α@Cur-MN patch significantly improved recovery of cardiac function, attenuated fibrosis, enhanced M2 macrophage infiltration, and promoted mature neovessel formation.

**Conclusion:**

This dual-target MN system provides a coordinated approach to regulating inflammation and angiogenesis, demonstrating therapeutic potential for myocardial repair.

**Graphical abstract:**

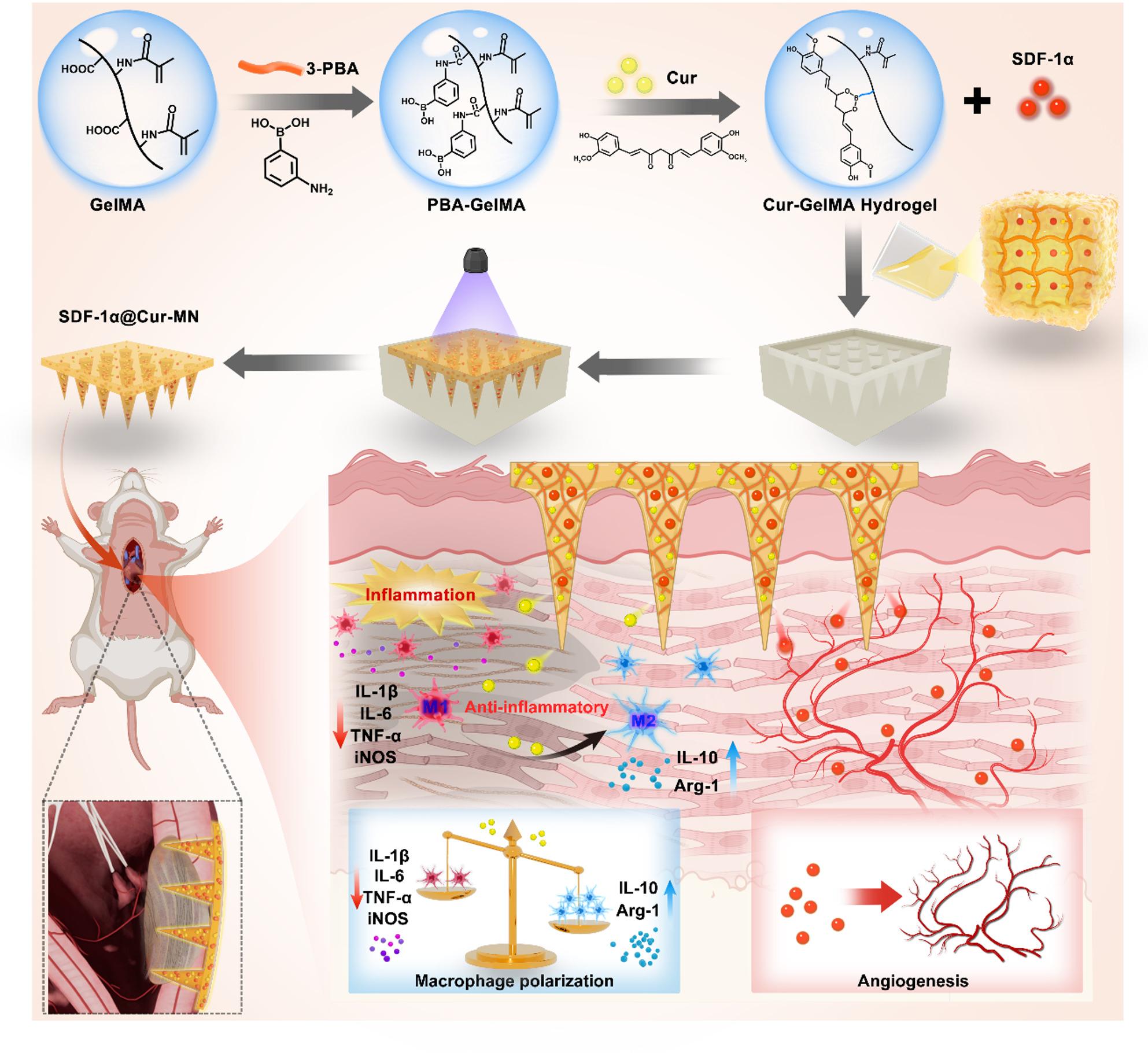

**Supplementary Information:**

The online version contains supplementary material available at 10.1186/s12951-026-04473-4.

## Introduction

Ischemic heart disease (IHD), the leading global cause of mortality, presents a major therapeutic challenge due to the irreversible necrosis and progressive ventricular remodeling triggered by myocardial infarction (MI) [1, 2]. Despite symptom-focused drugs (angiotensin-converting enzyme inhibitors, angiotensin receptor blockers, β-blockers), and revascularization, clinical interventions fail to modulate the pathological infarct microenvironment [3, 4]. Specifically, the pathological MI progression progresses through three overlapping phases: inflammation, proliferation, and remodeling [5]. Current therapeutic strategies primarily target four key processes: inhibiting cardiomyocyte death, suppressing inflammation, reversing fibrosis, and promoting angiogenesis [6]. However, these strategies mostly target a single pathological process, and lack spatiotemporal control over the microenvironment in the infarcted area. Consequently, integrated delivery systems capable of concurrently suppressing inflammatory cascades and orchestrating tissue regeneration represent a pivotal breakthrough strategy to overcome myocardial regeneration barriers.

Post-MI inflammation is initially essential for necrotic debris clearance, yet its transition into a persistent, M1 macrophage-dominated status acts as a primary driver of pathological cascades [7, 8]. CCL2/MCP-1-mediated monocytes infiltrate the infarct, differentiating into pro-inflammatory M1 macrophages (CD86⁺/iNOS⁺) that sustain TNF-α/IL-6 cytokine storms. This suppresses M2 reparative macrophages (CD206⁺/Arg-1⁺) polarization, blocking inflammation resolution. Consequent MMP-9-mediated fibroblast activation exacerbates collagen deposition, accelerating ventricular dilation and systolic dysfunction [9, 10]. Therefore, precise macrophage reprogramming represents a promising strategy to alleviate the inflammatory microenvironment and promote cardiac repair after MI [11, 12]. Curcumin (Cur), a natural polyphenol, has emerges as a potent regulator for modulating macrophage polarization via NF-κB inhibition/Nrf2 activation to resolve inflammation and remodel the microenvironment [13]. However, its clinical translation is restricted by its extreme hydrophobicity (0.6 µg/mL solubility) [14], rapid metabolism (oral bioavailability < 1%, half-life t₁/₂ ~5 min) [15], and non-specific distribution (myocardial accumulation < 0.5%) [16]. While traditional delivery fails, advanced nano-carriers (liposomes, micelles) and hydrogels offer promising alternatives to enhance the loading efficiency and bioavailability of curcumin in recent years [17, 18].

As the progressing of MI, robust angiogenesis extending from the border zone to the infarct core is crucial for cardiac repair. Coordinated angiogenesis enables to reduce fibrotic scarring, inhibit adverse ventricular remodeling, and maintain cardiac function [19]. Growth factor therapy is widely utilized to promote post-MI angiogenesis and involves various factors such as vascular endothelial growth factor (VEGF), basic fibroblast growth factor (bFGF), hepatocyte growth factor (HGF), and stromal cell-derived factor 1α (SDF-1α) [20–23]. These growth factors facilitate the restoration of functional microcirculation in infarcted zone through re-establishing perfusion. Among these angiogenic factors, SDF-1α offers unique advantages for myocardial repair. Unlike VEGF or PDGF, it possesses dual functionality of directly stimulating angiogenesis via CXCR4 signaling while recruiting circulating CXCR4-positive progenitor cells to the injury site [24–26]. Furthermore, the SDF-1α/CXCR4 axis amplifies multiple downstream pathways including VEGF upregulation, orchestrating a broader angiogenic program that addresses the limitation of single-factor therapies [27]. However, direct injection of SDF-1α is hindered by poor targeting, enzymatic inactivation and rapid degradation, severely limiting therapeutic efficacy [28]. To address these limitations, hydrogel-based delivery system provides a solution for controlled and sustained release of SDF-1α [29, 30]. For example, heparin/hyaluronic acid hydrogels sustained SDF-1α release for 14 days via electrostatic protection [31, 32].

Hydrogels, as localized delivery platforms, effectively overcome direct cardiac drug delivery limitations including low utilization, poor targeting, and metabolic instability [33–35]. Current hydrogel systems for cardiac repair comprise injectable hydrogels, cardiac adhesive patches, and microneedle patches [36–38]. Injectable hydrogels allow minimally invasive implantation but suffer rapid clearance and poor drug retention. Cardiac adhesive patches enable direct epicardial intervention and establish a protective niche that enhances cell retention and survival; however, their penetration depth remains limited. In contrast, microneedle (MN) patches achieve uniform epicardial penetration through micron-scale tips (e.g., 800 μm height), ensuring sustained drug release with broad myocardial distribution while avoiding injection-site accumulation [39–41]. MN patches delivering gene therapeutics, exosomes, growth factors, biofunctional peptides, and stem cells demonstrate therapeutic efficacy in ischemic cardiomyopathy [41–45]. For instance, adeno-associated virus serotype 9 (AAV-9)-loaded MN patches leverage stable cardiac affinity for efficient gene delivery [46]. Such patches also accommodate nanoparticle/liposome carriers for controlled release [47, 48].

**Scheme 1:**
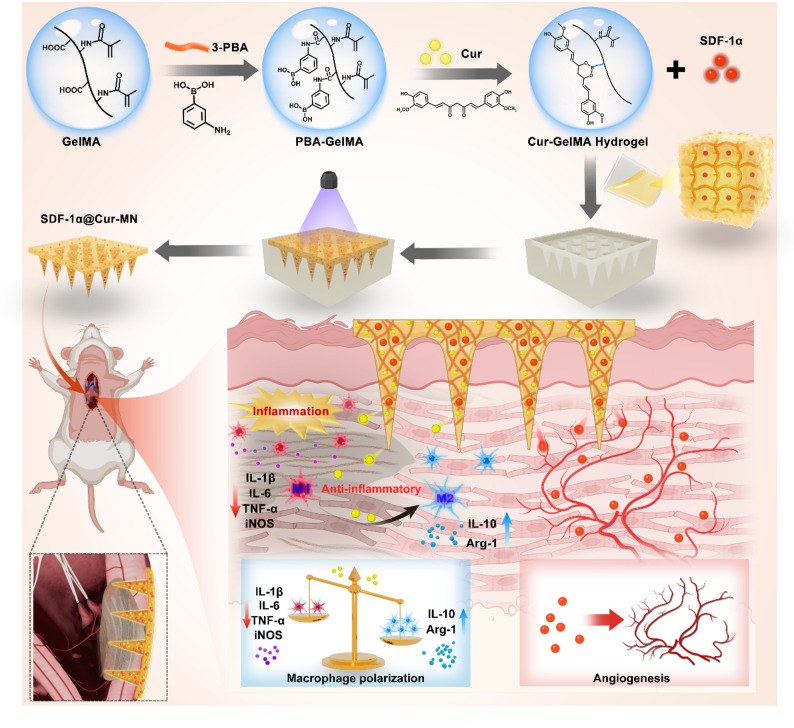
Schematic diagram of the curcumin hydrogel-based microneedle patch encapsulating SDF-1α (SDF-1α@Cur-MN) for myocardial infarction treatment. Curcumin hydrogel suppresses inflammatory responses by inducing macrophage polarization towards the M2 phenotype, synergizing with the angiogenic effect of SDF-1α to treat myocardial infarction

Herein, we developed an integrated microneedle (MN) patch based on a curcumin-conjugated hydrogel for co-delivery of curcumin and SDF-1α (denoted as SDF-1α@Cur-MN), aiming to concurrently suppress inflammatory cascades and promote angiogenesis after MI (Scheme 1). The curcumin hydrogel was synthesized by covalently conjugating curcumin onto gelatin methacrylate (GelMA) scaffolds, forming a dynamically crosslinked network that significantly improved curcumin’s solubility and bioavailability. The hydrogel was subsequently fabricated into SDF-1α-loaded MN patch via PDMS micromolding. The resulting SDF-1α@Cur-MN patch enabled rapid release of curcumin with sustained SDF-1α delivery, achieving spatiotemporally programmed drug release. The MN-based approach ensured targeted penetration into the infarcted region, facilitating localized high-concentration delivery while minimizing systemic exposure and overcoming targeting and utilization challenges. In vitro, curcumin hydrogel effectively regulated macrophage polarization, shifting the M1:M2 ratio from 3.4:1 to 0.7:1, downregulating proinflammatory cytokines (IL-1β, TNF-α, IL-6) and upregulating anti-inflammatory cytokine IL-10. Meanwhile, sustained SDF-1α release promoted endothelial proliferation and migration via activation of the VEGF pathway. In a rat MI model, treatment with SDF-1α@Cur-MN significantly improved the recovery of cardiac function and attenuated fibrosis, along with enhanced M2 macrophage infiltration and elevated density of mature neovessels in the infarct zone. This dual-target MN system represents a spatiotemporally coordinated strategy to synchronously regulate anti-inflammatory remodeling and angiogenic activation, supporting its potential application in myocardial repair.

## Materials and methods

### Preparation of curcumin-grafted GelMA hydrogel

Curcumin-grafted GelMA (denoted as Cur-GelMA) was prepared via a two-step conjugation strategy. First, 3-aminophenylboronic acid (3-PBA) was covalently coupled to GelMA through EDC/NHS-mediated amide bond formation between the amino group of 3-PBA and carboxyl groups on GelMA, yielding PBA-modified GelMA (denoted as PBA-GelMA). The curcumin was subsequently conjugated to PBA-GelMA via formation of phenylboronic acid ester bonds. Briefly, a total of 2.0 g methacrylated gelatin (GelMA; EFL-GM-30, Suzhou Yongqinquan Intelligent Equipment Co., Ltd.) was dissolved in 50 mL phosphate-buffered saline (PBS, pH 5.5). In parallel, 3-PBA (5.0 g; Sigma-Aldrich), N-hydroxysuccinimide (NHS; 1.5 g; Sigma-Aldrich), and 1-ethyl-3-(3-dimethylaminopropyl)carbodiimide hydrochloride (EDC·HCl; 4.0 g; Sigma-Aldrich) were dissolved in PBS and incubated at 4 °C for 6 h. The two solutions were then combined and stirred at 60 °C for 48 h. The resulting product was purified by dialysis and lyophilized to yield PBA-GelMA. The structure was characterized by ^1^H nuclear magnetic resonance (^1^H NMR; AVANCE III 400 M, Bruker, U.S.A.) and Fourier transform infrared spectroscopy (FTIR, TENSOR II, Bruker, U.S.A.).

For curcumin conjugation, PBA-GelMA (20 mg) was dissolved in 2 mL PBS (pH 5.5), followed by dropwise addition of a curcumin solution (2 mg/mL in DMSO; MedChemExpress LLC). The mixture was stirred at room temperature for 12 h in the dark. As a result, curcumin was subsequently loaded onto the PBA-GelMA hydrogel via the formation of phenylboronic acid ester bonds. Unbound curcumin was removed by dialysis, and the Cur-GelMA was obtained by lyophilization. The interaction between curcumin and GelMA was further evaluated by zeta potential analysis. Curcumin content in Cur-GelMA was quantified using a UV-Vis spectrophotometer (UV2600, Shimadzu, Japan) at 425 nm. The drug loading content (DLC) was calculated as DLC (%) = (mass/weight of curcumin) / (mass/ weight of Cur-GelMA) × 100%.

### Rheological characterization of hydrogels

The viscoelastic properties of GelMA, PBA-GelMA, and Cur-GelMA hydrogels were characterized using a Discovery Hybrid Rheometer HR30 (TA Instruments, New Castle, DE, USA). The hydrogels were molded into small cylinders measuring 8 mm in diameter with a volume of 150 µL. Oscillatory time sweep tests were performed at 1% strain and 1.0 Hz to determine the storage and loss moduli. Subsequently, oscillatory frequency sweeps were conducted at 1% strain over a frequency range of 0.1 Hz to 100 Hz.

### Preparation of SDF-1α-loaded microneedle patches

Microneedle (MN) molds were designed with a needle height of 800 μm and fabricated using a three-dimensional (3D) printer (nanoArch S140, BMF Precision Tech Inc., China) (Figure S1). The printed molds were subsequently replicated using polydimethylsiloxane (PDMS; SYLGARD™ 184 Silicone Elastomer, USA), which served as the template for MN patch fabrication. To prepare the blank MN patches, GelMA or Cur-GelMA was dissolved in PBS containing lithium phenyl-2,4,6-trimethylbenzoylphosphinate (LAP; 2.5 mg/mL) at 40 °C to prepare a 20% (w/v) prepolymer solution. An initial 200 µL aliquot was dispensed into the PDMS mold and centrifuged at 3000 rpm for 3 min to remove air bubbles, followed by addition of an additional 300 µL of prepolymer. The filled molds were dried at 37 °C for 12 h and then exposed to ultraviolet light for 60 s to complete polymerization. After curing and drying, the MN arrays were demolded to obtain GelMA MN patches (denoted as G-MN) or Cur-GelMA MN patches (denoted as Cur-MN).

For preparation of the SDF-1α-loaded MN patches, 3 µg of SDF-1α protein (Thermo Fisher Scientific) was incorporated into the 20% (w/v) GelMA or Cur-GelMA prepolymer solution. Each mixture was then cast into a PDMS mold and fabricated using the above procedure, to obtain SDF-1α-loaded GelMA MN patch (denoted as SDF-1α@G-MN) or SDF-1α-loaded Cur-GelMA MN patch (denoted as SDF-1α@Cur-MN).

### Characterization of microneedle patches

The morphology and surface structure of MN patches were examined using a handheld digital microscope (AF4515ZT4, AnMo Electronics Corporation, Taiwan) and scanning electron microscopy (SEM; TM4000Plus II, Hitachi, Japan), respectively. Mechanical properties were assessed using a texture analyzer (TA.XTC-20, Shanghai Baosheng Industrial Development Co., Ltd., China). Briefly, the microneedles were mounted vertically on the test platform with the tips facing upward. A force sensor was then used to measure the mechanical strength of the needle tips. For testing penetration tissue capability by MN, red fluorescence dye, Rhodamine B (RhB) was incorporated into the Cur-GelMA hydrogel for fabrication of RhB-loaded Cur-MN patch. The MN patch was applied to freshly excised rat left ventricular myocardium with gentle manual pressure, then removed. Penetration into cardiac tissue was assessed by 3D scanning using laser scanning confocal microscopy (LSCM).

### Evaluation of tissue adhesion strength

The adhesion strength of the MN patch to cardiac tissue was evaluated using a mechanical testing system (MTS Criterion Model 43, USA) equipped with a 10 N load cell. Freshly excised rat hearts were obtained immediately after euthanasia and maintained moist with PBS throughout the experiment. The heart was securely positioned on the lower platform of the testing apparatus, and the Cur-MN patch was gently applied to the epicardial surface with consistent manual pressure for 30 s to ensure initial contact and MN insertion. The patch was then pulled vertically upward at a constant speed of 1 mm/s, and the force-displacement curve was recorded until complete detachment. The maximum detachment force (*F*_max_) was determined from the curve and normalized to the contact area (A) to calculate the adhesive strength using the formula: Adhesive strength (kPa) = *F*_max_ / A. As a control, a bulk hydrogel patch of identical hydrogel and dimensions but without microneedle structures was tested using the same procedure.

### Drug release of SDF-1α@Cur-MN patch

To simulate the release behavior of stromal cell-derived factor-1α (SDF-1α), lysozyme (LZ; Guangzhou Magen Biotechnology Co., Ltd.), a protein with a comparable molecular weight, was used as a substitute in in vitro release studies. Prior to the experiment, standard calibration curves for curcumin (Cur) and LZ were established using dimethyl sulfoxide (DMSO) and PBS as solvents by serial dilution and spectrophotometric analysis, respectively. To assess the release kinetics of Cur and SDF-1α from the microneedle patches, the prepared formulations were placed in the Franz diffusion cells maintained at 37 °C. To evaluate the ROS-responsive release characteristics, two different release media were employed: (1) PBS (pH = 7.4) to simulate normal physiological conditions, and (2) PBS (pH = 5.5) containing 200 µM H_2_O_2_ to mimic the acidic and oxidative microenvironment of infarcted myocardium. On the first day, 100 µL of release medium was collected at hourly intervals, and the absorbance of each sample was measured spectrophotometrically (425 nm for Cur and 280 nm for LZ). After each sampling, an equivalent volume of fresh PBS was added to maintain a constant volume. From the second day onward, sampling was conducted daily using the same protocol. The cumulative release profiles of Cur and SDF-1α from the SDF-1α@Cur-MN patches were calculated based on the absorbance measurements and corresponding standard curves.

### Evaluation of in vitro and in vivo degradation of the MN patch

The in vitro degradation behavior of the SDF-1α@Cur-MN patch was evaluated by monitoring mass loss over time. Briefly, freshly prepared patches were weighed (initial weight, W_o_) and then incubated in PBS (pH 7.4) at 37 °C with gentle shaking (50 rpm). At predetermined time points (days 0, 4, 8, 12, 16, and 20), the patches were carefully removed, rinsed with deionized water to remove buffer salts, lyophilized, and reweighed (W_t_). The percentage of mass remaining was calculated using the formula: Mass remaining (%) = (W_t_ / W_o_) × 100%. All measurements were performed in triplicate.

To assess in vivo degradation profile, SDF-1α@Cur-MN patches were implanted onto the epicardial surface of rat hearts immediately following left thoracotomy. At 2-, 3-, 6-, 9-, 10-, 12, and 15-days post-implantation, rats were euthanized, and the hearts were excised. The implantation sites were carefully examined, and the residual patches were photographed using a stereomicroscope to visualize the extent of degradation.

### Cell culture

The rat cardiomyocyte cell line (H9C2) was maintained in Dulbecco’s Modified Eagle Medium (DMEM; Gibco, USA) supplemented with 10% fetal bovine serum (FBS; ExCell Bio, China). Meanwhile, the mouse macrophage cell line (RAW 264.7) was cultured in DMEM (Procell, China) supplemented with 10% FBS (ExCell Bio, China). Human umbilical vein endothelial cells (HUVECs) were cultured in Endothelial Cell Medium (ScienCell, USA) supplemented with 10% FBS (ScienCell, USA). All the cells were incubated at 37 °C in a humidified atmosphere containing 5% CO_2_.

### Cell viability assay

For in vitro biocompatibility of the microneedle patches, the H9C2 cardiomyocytes were seeded into 12-well plates at a density of 1 × 10^5^ cells/well. After 12 h, the culture medium was replaced with DMEM containing extracts from the microneedle patches for 24 and 48 h, respectively. Cell viability was subsequently assessed using a Live/Dead staining kit, according to the manufacturer’s protocol. Briefly, the H9C2 cardiomyocytes were first fixed with 4% paraformaldehyde, and then subjected to double staining with calcein AM and propidium iodide (Beyotime, China) for 30 min at room temperature. To assess cell viability, the cells were imaged using a confocal laser scanning microscope (LSM880, Carl Zeiss). The proportion of live cells was used to determine the cytocompatibility of the microneedle-derived materials.

### CCK-8 assay

The cell viability of H9C2 cardiomyocytes and the proliferation rate of HUVECs following treatment with extracts from various microneedle (MN) patches were assessed using the Cell Counting Kit-8 (CCK-8; Dojindo, Japan). In brief, H9C2 and HUVECs cells were seeded into 96-well plates at a density of 8 × 10^3^ cells/well and incubated for 12 h. Following incubation, the culture medium was replaced with medium containing extracts from different MN patches corresponding to experimental groups. For H9C2 cardiomyocytes, 10 µL of CCK-8 reagent was added to each well and co-cultured for 24 and 48 h. For HUVECs, 10 µL of CCK-8 reagent was added and co-cultured at 24 h. The detection of optical density (OD) at 450 nm was measured using a microplate reader to quantify cell viability and proliferation.

### Flow cytometry analysis of macrophage polarization

Flow cytometry was utilized to assess the effects of extracts derived from microneedle (MN) patches on macrophage polarization. RAW 264.7 murine macrophages were seeded in 6-well plates at a density of 1 × 10^6^ cells/well and cultured in the presence or absence of 100 ng/mL of lipopolysaccharide (LPS) to induce an inflammatory response (Figure S7). After 24 h, the culture medium was replaced with medium containing extracts from MN patches corresponding to the designated experimental groups. Following a 24-h incubation, the cells were harvested and stained with fluorescence-conjugated monoclonal antibodies: phycoerythrin (PE)-conjugated anti-mouse CD86 (Elabscience, E-AB-F0994D) to identify M1 macrophages, and allophycocyanin (APC)-conjugated anti-mouse CD206 (Elabscience, E-AB-F1135E) to identify M2 macrophages. Samples were analyzed on a flow cytometer, and the expression levels of polarization-associated markers were quantified to determine macrophage phenotype distribution.

### Quantitative real-time polymerase chain reaction (RT-qPCR)

Total RNA was extracted from RAW 264.7 cells using TRIzol reagent (Accurate Biotechnology, China), and RNA concentrations were quantified using a NanoDrop spectrophotometer (Thermo Fisher Scientific Inc., USA). Complementary DNA (cDNA) was synthesized from the extracted RNA through reverse transcription using a cDNA synthesis kit (Accurate Biotechnology, China) according to the manufacturer’s instructions. Quantitative real-time PCR (RT-qPCR) was conducted using the LightCycler 480 II system (Roche, USA) with SYBR Green PCR Master Mix (Accurate Biology, China). The relative mRNA expression levels of cytokines, including IL-1β, IL-6, TNF-α, and IL-10, were determined using the 2^–ΔΔCT^ method and normalized to the GAPDH housekeeping gene.

### Western blotting

Total protein lysates were prepared from RAW 264.7 and HUVECs cell samples using RIPA lysis buffer (Beyotime, China) supplemented with protease and phosphatase inhibitor cocktails (Thermo Fisher Scientific, USA). Protein concentrations were measured by bicinchoninic acid (BCA) assay. A total of 20 µg protein was loaded onto sodium dodecyl sulfate-polyacrylamide gels (SDS-PAGE, Beyotime, China) for electrophoretic separation. Following electrophoresis, proteins were transferred onto polyvinylidene fluoride (PVDF) membranes (Millipore, USA) and blocked with 5% skim milk in Tris-buffered saline containing 0.1% Tween-20 (TBST) for 1 h at room temperature. The membranes were then incubated overnight at 4 °C with the following primary antibodies: β-actin (Cell Signaling Technology, #4967), iNOS (Proteintech, 22226-1-AP), Arg-1 (Proteintech, 16001-1-AP), GAPDH (Cell Signaling Technology, #5174), and VEGF (Abcam, ab46154). After washing with TBST, the membranes were incubated with horseradish peroxidase-conjugated anti-rabbit IgG secondary antibody (Cell Signaling Technology, #7074) for 1 h at room temperature. Immunoreactive bands were detected using an enhanced chemiluminescence (ECL) detection system and captured using an Amersham Imager 600 (GE Healthcare, USA). The density of protein bands was quantified using ImageJ software. The relative expression levels of target proteins were eventually normalized to β-actin or GAPDH.

### Scratch wound migration assay

Human umbilical vein endothelial cells (HUVECs) were seeded in 6-well plates at a density of 2 × 10^5^ cells/well and cultured for 24 h to allow formation of a confluent monolayer. A linear wound was generated by gently scratching the monolayer with a sterile 200 µL pipette tip. Detached cells were removed by rinsing with PBS, after which cells were cultured in complete medium containing extracts from the designated MN patches. Phase-contrast images of the wound area were captured at 0, 12, 24, and 36 h post-treatment using an inverted microscope. The wound closure was quantified by measuring the residual wound area at each time point relative to the initial wound area using ImageJ software. The percentage of wound closure was calculated to evaluate cell migratory capacity.

### Endothelial tube formation assay

Matrigel (Corning, 354234) was thawed on ice, and 50 µL aliquots were dispensed into pre-chilled 96-well plates. The Matrigel was allowed to polymerize at 37 °C for 30 min. HUVECs were then resuspended at 1 × 10^4^ cells/well in 100 µL of culture medium containing the extract from the designated MN patches. The cell suspension was gently overlaid onto the Matrigel-coated plate and incubated at 37 °C in a humidified atmosphere with 5% CO_2_ for 6 h. Tubular network formation was visualized and recorded using a microscopy. Quantitative analysis of total tube length and number of branch points was performed using the angiogenesis analysis plugin in ImageJ software.

### Establishment of myocardial infarction rat model and implantation of MN patch

All experimental procedures were approved by the Animal Research Committee of Guangzhou Medical University (No. GY2024-462) and performed in accordance with the National Institutes of Health (NIH) guidelines for the care and use of laboratory animals. Male Sprague-Dawley (SD) rats (6 weeks old, 220–250 g) were purchased from Beijing Weitong Lihua Experimental Animal Technology Co., Ltd. and housed under standard laboratory conditions at the animal facility of Guangzhou Medical University, with ad libitum access to food and water.

Animals were randomly assigned to six experimental groups: Sham (*n* = 5), Myocardial infarction (MI, *n* = 6), G-MN (MI treated with G-MN patches, *n* = 5), Cur-MN (MI treated with Cur-MN patches, *n* = 6), SDF-1α@G-MN (MI treated with SDF-1α@G-MN patches containing 3 µg of SDF-1α, *n* = 6), SDF-1α@Cur-MN (MI treated with SDF-1α@Cur-MN containing 3 µg of SDF-1α, *n* = 7). According to DLC of Cur-GelMA hydrogel, each MN patch was formulated with 1000 µg of curcumin.

To establish the MI model, rats were anesthetized via intraperitoneal injection of pentobarbital sodium (40 mg/kg), followed by endotracheal intubation and mechanical ventilation with a small animal ventilator (Kent Scientific Corporation). A left thoracotomy was performed between the third and fourth intercostal spaces, and the pericardium was carefully removed to expose the heart. The left anterior descending (LAD) coronary artery was permanently ligated using 8 − 0 Prolene sutures. Successful occlusion was confirmed by the immediate appearance of myocardial blanching and apical cyanosis. After LAD ligation, the designated microneedle patches were implanted directly onto the infarcted region of the myocardium.

### Echocardiographic assessment of cardiac function

Cardiac function was evaluated using a Vevo 3100 high-resolution ultrasound imaging system, equipped with a 30-MHz ultrasound transducer (VisualSonics, Canada). In brief, rats were anesthetized via inhalation of 2% isoflurane and positioned on the surgical board for stable imaging. Two-dimensional (B-mode) and motion-mode (M-mode) echocardiographic images were acquired from both the long and short axes of the left ventricular (LV) at days 7, 14, and 28 post-MI. Image acquisition was conducted using standardized parasternal views. Quantitative analysis of the echocardiographic images was performed using Vevo LAB 5.8.2 software (VisualSonics). Cardiac parameters were calculated to assess left ventricular systolic function, including ejection fraction (EF), fractional shortening (FS), end-systolic volume (ESV), and end-diastolic volume (EDV). For each measurement, data from three consecutive cardiac cycles were recorded and averaged to ensure accuracy.

### Histology and immunohistochemistry

At days 7 and 28 post-MI, rats from each experimental group were euthanized for tissue collection. The heart samples were excised and thoroughly rinsed with cold saline to remove residual blood. Approximately 20 mg of cardiac tissue from the infarct zone was dissected and immediately stored in liquid nitrogen for subsequent total RNA extraction and RT-qPCR analysis of cytokines expression, including IL-6 and IL-10.

The remaining cardiac tissue was fixed in 4% paraformaldehyde, embedded in paraffin, and sectioned for histological and immunohistochemical analysis. Hematoxylin and eosin (H&E) staining was conducted to assess myocardial morphology, while Masson’s trichrome staining was performed to evaluate collagen deposition and myocardial fibrosis. To examine macrophage polarization, immunofluorescence staining was performed using antibodies against CD86 (M1 macrophage marker, Proteintech, 26903-1-AP) and CD206 (M2 macrophage marker, Proteintech, 18704-1-AP). Inflammatory cell infiltration was assessed by immunofluorescence staining for TNF-α (Abcam, ab6671). Apoptotic cells within the infarct region were detected using a terminal deoxynucleotidyl transferase dUTP nick-end labeling (TUNEL) apoptosis detection kit (Elabscience, China). Cardiomyocyte proliferation was assessed by immunostaining for Ki67 (Servicebio, GB111499). In addition, vascularization and smooth muscle cell distribution were examined through immunofluorescence staining for CD31 (Abcam, ab281583) and α-SMA (Servicebio, GB111364), respectively.

### Enzyme-Linked Immunosorbent Assay (ELISA)

At day 7 post-MI, approximately 2 mL of whole blood was collected from each rat via tail amputation. The blood samples were allowed to clot at room temperature for 2 h and subsequently centrifuged at 3000 × *g* for 15 min at 4 °C using a refrigerated centrifuge (Thermo Fisher Scientific, USA). The resulting serum was carefully collected for further analysis. Cytokine concentrations of IL-1β, IL-6, and IL-10 were quantified using ELISA kits (Neobioscience, China), according to the manufacturer’s instructions.

### Statistical analysis

All quantitative data were presented as the means ± standard error of the mean (SEM). Statistical comparison between the two groups was performed using a two-tailed unpaired Student’s t-tests. For comparisons among multiple groups, one-way analysis of variance (ANOVA) followed by Tukey’s multiple comparisons test was applied. A *p* value < 0.05 was considered statistically significant. Significance levels are indicated as follows: *p* < 0.05 (*), *p* < 0.01 (**), *p* < 0.001 (***), and *p* < 0.0001 (****), whereas “ns” denotes non-significance.

## Results

### Preparation and characterization of curcumin-grafted GelMA hydrogel

**Fig. 1:**
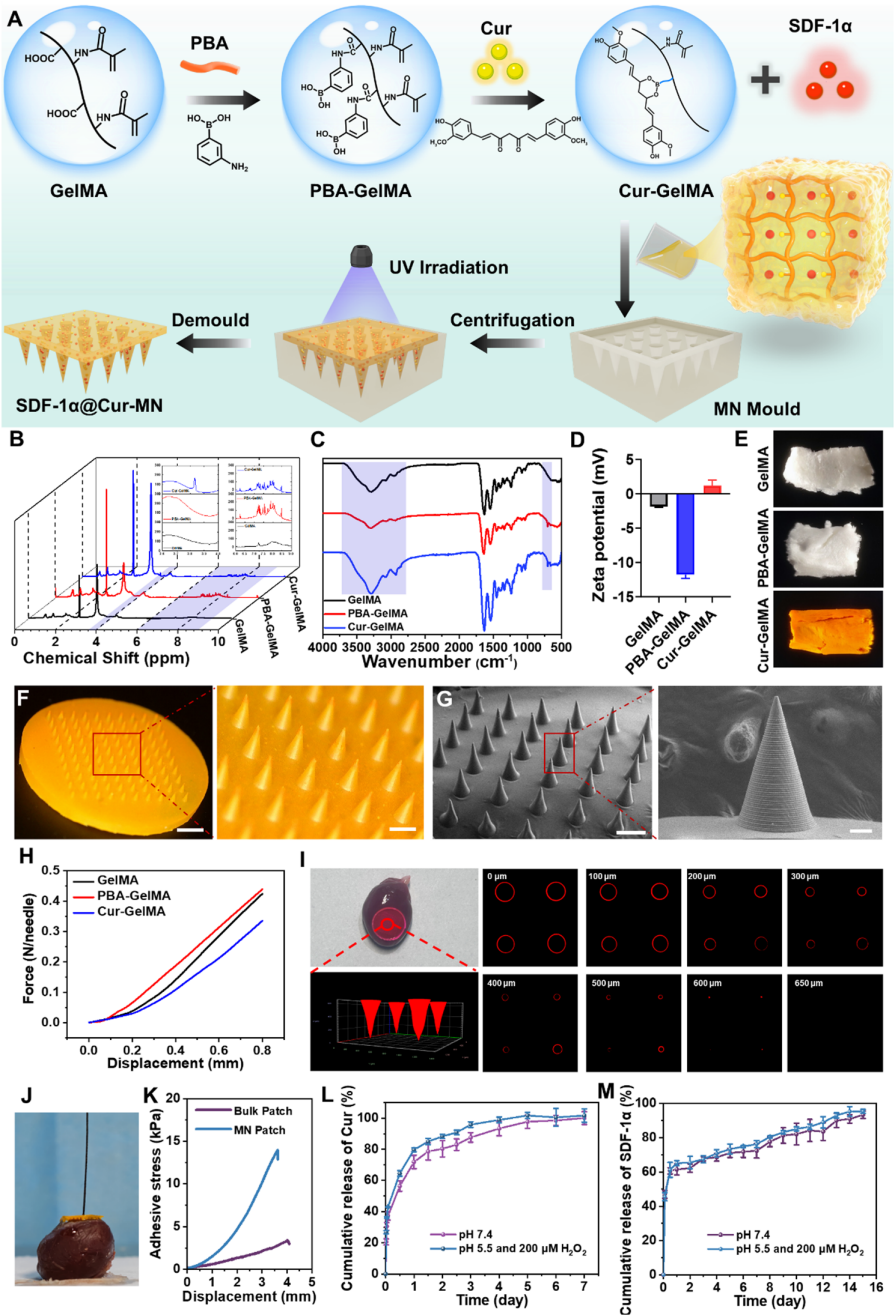
Preparation and characterization of curcumin hydrogel and the microneedle patch. **(A)** Schematic diagram illustrating the procedure for conjugating GelMA with curcumin and fabricating the curcumin hydrogel-based microneedle patch. **(B)** ^1^H NMR spectra of GelMA, PBA-GelMA, and Cur-GelMA. **(C)** FTIR spectra of GelMA, PBA-GelMA, and Cur-GelMA. **(D)** Zeta potential measurements of GelMA, PBA-GelMA, and Cur-GelMA. **(E)** Photographs of different hydrogel materials. **(F)** Photographs of the SDF-1α@Cur-MN patch. Scale bars: 2000 μm (overview) and 200 μm (magnified view). **(G)** SEM images of the microneedle patch. Scale bars: 500 μm (overview) and 100 μm (magnified view). **(H)** Mechanical strength test of the microneedle patches fabricated from GelMA, PBA-GelMA, and Cur-GelMA. (**I**) Confocal microscopy images illustrating the morphology and penetration depth of the Rhodamine B-loaded Cur-GelMA MN patch after puncturing cardiac tissue. Representative images showing the morphology of the MN tips. Fluorescence images depicting the spatial distribution of Rhodamine B within cardiac tissue, indicating the penetration depths of individual microneedles. **(J)** Representative photograph of the ex vivo adhesion test setup, showing the SDF-1α@Cur-MN patch attached to the epicardial surface of a rat heart and connected to the mechanical testing system. **(K)** Force-displacement curves of adhesive strength between the SDF-1α@Cur-MN patch and a bulk hydrogel patch without microneedle structures. **(L**,** M)** In vitro cumulative release profiles of curcumin **(L)** and SDF-1α **(M)** from the microneedle patches under two different conditions: PBS (pH = 7.4), and PBS (pH = 5.5) containing 200 µM H_2_O_2_ (*n* = 6)

Due to the superior biocompatibility, mechanical strength, and biodegradability, GelMA was utilized to fabricate the MN patch as a carrier for co-delivery of curcumin and SDF-1α to the infarcted myocardium [49]. As illustrated in Fig. 1A, the synthesis of curcumin-grafted GelMA hydrogel (Cur-GelMA) was conducted via a two-step modification. First, 3-aminophenylboronic acid (3-PBA) was covalently grafted onto the GelMA scaffold through amide reaction between the amino groups of 3-PBA and the carboxyl groups of GelMA. Subsequently, curcumin was conjugated via phenylboronic acid ester bonds. This approach not only markedly enhanced curcumin loading efficiency but also endowed the hydrogel with reactive oxygen species (ROS)-responsive release properties, enabling rapid curcumin liberation to facilitate early anti-inflammatory intervention in the high-ROS microenvironment associated with myocardial infarction (MI). To confirm successful 3-PBA and curcumin grafting, the chemical structures of GelMA, PBA-modified GelMA (PBA-GelMA), and Cur-GelMA hydrogel were analyzed by ¹H nuclear magnetic resonance (¹H NMR) and Fourier transform infrared spectroscopy (FTIR). As shown in Fig. 1B, compared with the ¹H NMR spectrum of unmodified GelMA, PBA-GelMA exhibited new peaks between 7.0 and 8.5 ppm corresponding to aromatic protons in the benzene ring, indicative of successful PBA incorporation and consistent with previous literature [50]. The substitution degree of PBA was determined to be approximately 53.2%. Furthermore, upon curcumin conjugation, the ¹H NMR spectrum of Cur-GelMA revealed several newly emerged proton signals that can be unambiguously assigned to curcumin. Specifically, a set of aromatic proton signals appears as multiplets in the region of δ 7.0–8.5 ppm, corresponding to the benzene rings of curcumin. A sharp singlet at approximately δ 6.0 ppm was attributed to the olefinic proton (= C–H) of the heptadienone linker bridging the two aromatic rings in curcumin. In addition, a distinct peak around δ 3.8–4.0 ppm was assigned to the protons of the methoxy groups (–OCH₃) on the curcumin aromatic rings, revealing that approximately 65% of the available PBA groups were successfully conjugated with curcumin. The presence of these characteristic signals provides clear evidence for the successful incorporation of curcumin into the PBA-GelMA network.

FTIR spectra revealed broad absorption bands between 3200 and 3500 cm⁻¹ in all hydrogel formulations, attributable to O–H and N–H stretching vibrations (Fig. 1C). Notably, PBA-GelMA displayed a distinct B–O stretching vibration at 1370 cm⁻¹ of boric acid, as well as C–H bending at 703 cm⁻¹ characteristic of benzene rings, confirming successful PBA modification. In the Cur-GelMA spectrum, the characteristic B–O stretching vibration (~ 1370 cm⁻¹) originating from the PBA linker was retained. More importantly, a prominent absorption band at approximately 1627 cm⁻¹ was observed, corresponding to the C = O stretching vibration of the β-diketone (enol) moiety of curcumin. The coexistence of the B–O vibration and the characteristic curcumin C = O peak, together with the appearance of C–H stretching vibrations around 2920 cm⁻¹ (attributed to the methyl and methylene groups of curcumin), strongly supports successful conjugation of curcumin into the PBA-GelMA. Collectively, these NMR and FTIR data provide robust and complementary evidence confirming the successful covalent grafting of curcumin onto the GelMA scaffold via the PBA linker.

Furthermore, the surface charge properties of the hydrogels were examined via zeta potential analysis using dynamic light scattering (DLS). As shown in Fig. 1D, unmodified GelMA exhibited a zeta potential of − 1.88 mV in ddH_2_O. After 3-PBA modification, the potential shifted to − 11.8 mV, indicating the presence of negatively charged borate anions at physiological pH. Upon curcumin conjugation, the zeta potential increased to + 1.20 mV, due to the formation of electrically neutral boronate ester bonds between PBA and curcumin, which reduced the overall negative charge contribution from borate anions. Besides, the optical images of GelMA, PBA-GelMA, and Cur-GelMA hydrogel was shown in Fig. 1E. The Cur-GelMA hydrogel exhibited a uniform yellow coloration, with no observable particulate aggregation or precipitation, indicative of homogeneous curcumin distribution. The drug loading content (DLC) was quantified by measuring the characteristic curcumin absorbance at 425 nm using UV–Vis spectroscopy, yielding a DLC value of 2.48% for the Cur-GelMA hydrogel. These findings collectively confirm the successful synthesis of curcumin-grafted GelMA hydrogel. Meanwhile, by conjugating curcumin onto the GelMA hydrogel, the effective loading capacity of curcumin can be significantly enhanced, thereby addressing its inherent poor water solubility.

The viscoelastic properties of GelMA, PBA-GelMA, and Cur-GelMA were characterized by rheological analysis. As shown in Figure S1A-B, all three hydrogels exhibited typical gel-like behavior, with storage modulus (G′) consistently exceeding loss modulus (G″) across the tested frequency range (0.1–100 Hz), confirming stable crosslinked network formation. Quantitative analysis at 1 Hz revealed that GelMA displayed G′ and G″ values of 2406 ± 9 Pa and 979 ± 8 Pa, respectively. Following PBA modification, PBA-GelMA showed a marked reduction in loss modulus (G″: 67 ± 3 Pa) while maintaining a comparable storage modulus (G′: 2256 ± 50 Pa). After curcumin conjugation, Cur-GelMA exhibited further decreased moduli, with G′ of 1034 ± 21 Pa and G″ of 24 ± 2 Pa. This progressive reduction, particularly after curcumin conjugation, is likely attributable to the introduction of bulky aromatic moieties that partially disrupt polymer chain packing during photo-crosslinking. Nevertheless, all hydrogels exhibited G′ values above 1 kPa, sufficient for maintaining microneedle structural integrity during fabrication and tissue penetration.

### Preparation and characterization of microneedle patches

To address the limitations of direct intramyocardial injection, a biodegradable microneedle (MN) patch was fabricated for myocardium infarction treatment. Cur-GelMA hydrogel was subsequently employed to fabricate MN patches for encapsulating SDF-1α via a centrifugation-moulding process (Fig. 1A). Briefly, SDF-1α was first mixed with Cur-GelMA hydrogel, injected into a microneedle mold containing an ordered array of conical cavities, and centrifuged to remove air bubbles. The arrays were then photo-crosslinked under UV light, followed by drying and demolding to obtain intact MN patches. As shown in Fig. 1F, the resulting Cur-GelMA MN patch exhibited a uniform geometry in a 10 × 10 array with intact tips. The entire patch including both the microneedle tips and the backing layer exhibited a uniform yellow coloration, indicating homogeneous distribution of the covalently conjugated curcumin throughout the hydrogel network. Scanning electron microscopy (SEM) analysis confirmed well-defined conical needles with an average height of 800 μm and a base diameter of 250 μm (Fig. 1G). For comparison, control MN patches made of GelMA and PBA-GelMA hydrogel, and RhB-loaded Cur-MN patches were prepared using the same process (Figure S3). Fluorescence microscopy revealed uniform red fluorescence across both the tips and the backing layer in the RhB-loaded Cur-MN patch (Figure S3D). Quantitatively, each patch contained approximately 1000 µg of Cur and 3 µg of SDF-1α. This homogeneous drug distribution is by design: the backing layer serves as a drug reservoir to ensure adequate single-dose payload, while the tips provide mechanical anchoring and create microchannels for enhanced drug penetration into the myocardial tissue.

The mechanical performance of the MN arrays was evaluated with a texture analyzer. As shown in Fig. 1H, force–displacement curves revealed minor variations among the different hydrogel formulations. All MN arrays deformed uniformly and showed no fracture at a maximum load of 10 N per patch, indicating sufficient mechanical robustness for cardiac tissue insertion while remaining compatible with epicardial tissue compliance. To further assess penetrability, Rhodamine B-loaded Cur-MN patches were manually applied to freshly excised cardiac tissue from Sprague-Dawley rat, followed by three-dimensional imaging using laser scanning confocal microscopy (LSCM). As shown in Fig. 1I, Z-stack confocal imaging revealed consistent intramyocardial delivery, with an apparent penetration depth of approximately 600 μm and strong red fluorescence distributed within the myocardium, demonstrating effective MN penetration into the myocardium and confirming feasibility for localized intramyocardial drug delivery.

To assess the retention capability of the MN patch on myocardial tissue under dynamic physiological condition, we performed ex vivo adhesion tests using a mechanical testing system. As shown in Fig. 1J, the experimental setup allowed direct measurement of the force required to detach the patch from the epicardial surface. The SDF-1α@Cur-MN patch exhibited a significantly higher adhesive strength of ~ 14 kPa, whereas the bulk patch without microneedle structures showed only ~ 3 kPa (Fig. 1K). This approximately 4.5-fold increase in adhesion strength can be attributed to the mechanical interlocking provided by the microneedle tips, which physically anchor into the myocardial tissue layer upon insertion. This anchoring mechanism is particularly critical for cardiac applications, as it enhances the patch’s resistance to detachment under the continuous and dynamic motion of the beating heart, thereby ensuring sustained drug delivery and therapeutic efficacy throughout the treatment period.

Release profiles of curcumin and SDF-1α from the MN patches were assessed using Franz diffusion cells under two different conditions: neutral pH without H_2_O_2_ (pH 7.4) and acidic oxidative conditions mimicking the MI microenvironment (pH 5.5 with 200 µM H_2_O_2_). Owing to the reactive oxygen species (ROS)- and acid-labile nature of the boronate ester bond between 3-PBA and curcumin, this linkage undergoes oxidative and hydrolytic cleavage in the presence of H_2_O_2_ and under mildly acidic conditions, leading to on-demand release of free curcumin [51]. As shown in Fig. 1L, curcumin exhibited markedly accelerated release under the acidic and oxidative condition. The cumulative release reached 42.9% within 2 h and 88.2% at 48 h under pH 5.5 with 200 µM H_2_O_2_, which was higher than the release observed under the neutral condition (37.2% at 2 h and 80.3% at 48 h). This enhanced release confirms the ROS-responsive characteristics of the boronate ester linkage, enabling rapid curcumin liberation in the high-ROS microenvironment of infarcted myocardium to facilitate early anti-inflammatory intervention [52]. As shown in Fig. 1M, the release profiles of SDF-1α showed no significant difference between the two conditions, with sustained release over two weeks. This lack of condition-dependent release is consistent with the fact that SDF-1α is physically encapsulated within the hydrogel matrix rather than covalently conjugated. Consequently, its release kinetics is primarily governed by passive diffusion and hydrogel degradation, which are largely unaffected by the relatively mild oxidative and acidic conditions. The sustained release of SDF-1α ensures its capacity for continuous delivery to promote myocardial repair and neovascularization during the subacute phase of MI. It should be noted that these in vitro release profiles represent a controlled comparative assessment of release kinetics rather than an absolute prediction of in vivo release rates. The distinct release behaviors, with ROS-responsive release for curcumin and sustained release for SDF-1α, not only reveal the underlying differential delivery mechanisms but also align with the therapeutic efficiency observed in vivo.

The in vitro biocompatibility of SDF-1α@Cur-MN patch was assessed by co-culturing with H9C2 cardiomyocytes for 1–2 days, followed by live-dead cell staining assays. No significant difference in green fluorescence of live cells was observed between the SDF-1α@Cur-MN patch-treated and control group over the 2-day period (Figure S4), confirming the excellent biocompatibility of SDF-1α@Cur-MN patch. In addition, to confirm the biodegradability of the SDF-1α@Cur-MN patch, we evaluated its degradation profiles both in vitro and in vivo. As shown in Figures S5A-B, the in vitro degradation study revealed that the patch underwent gradual mass loss when incubated in PBS at 37 °C. Consistent with the hydrolytic degradation kinetics of gelatin-based hydrogels, approximately 50% of the initial mass was lost by day 12, and the patch was nearly completely degraded (> 95% mass loss) by day 20.

We further investigated the in vivo degradation behavior of MN patches following epicardial implantation in a rat model. Macroscopic observation of the heart tissue at different time points post-implantation revealed a degradation trend consistent with the in vitro findings (Figure S6). At day 6 post-implantation, approximately half of the hydrogel patch remained visible on the epicardial surface. By day 15, the patch material was almost completely degraded, with only minimal residual fragments observed. By the endpoint of the treatment (day 28), no visible remnants were detectable, confirming the complete biodegradation and absorption of the patch in vivo. This degradation timeline aligns well with the therapeutic window required for sustained SDF-1α release and myocardial remodeling, ensuring that the patch doesn’t persist as a long-term foreign body.

### Effect of SDF-1α@Cur-MN on macrophages polarization

**Fig. 2:**
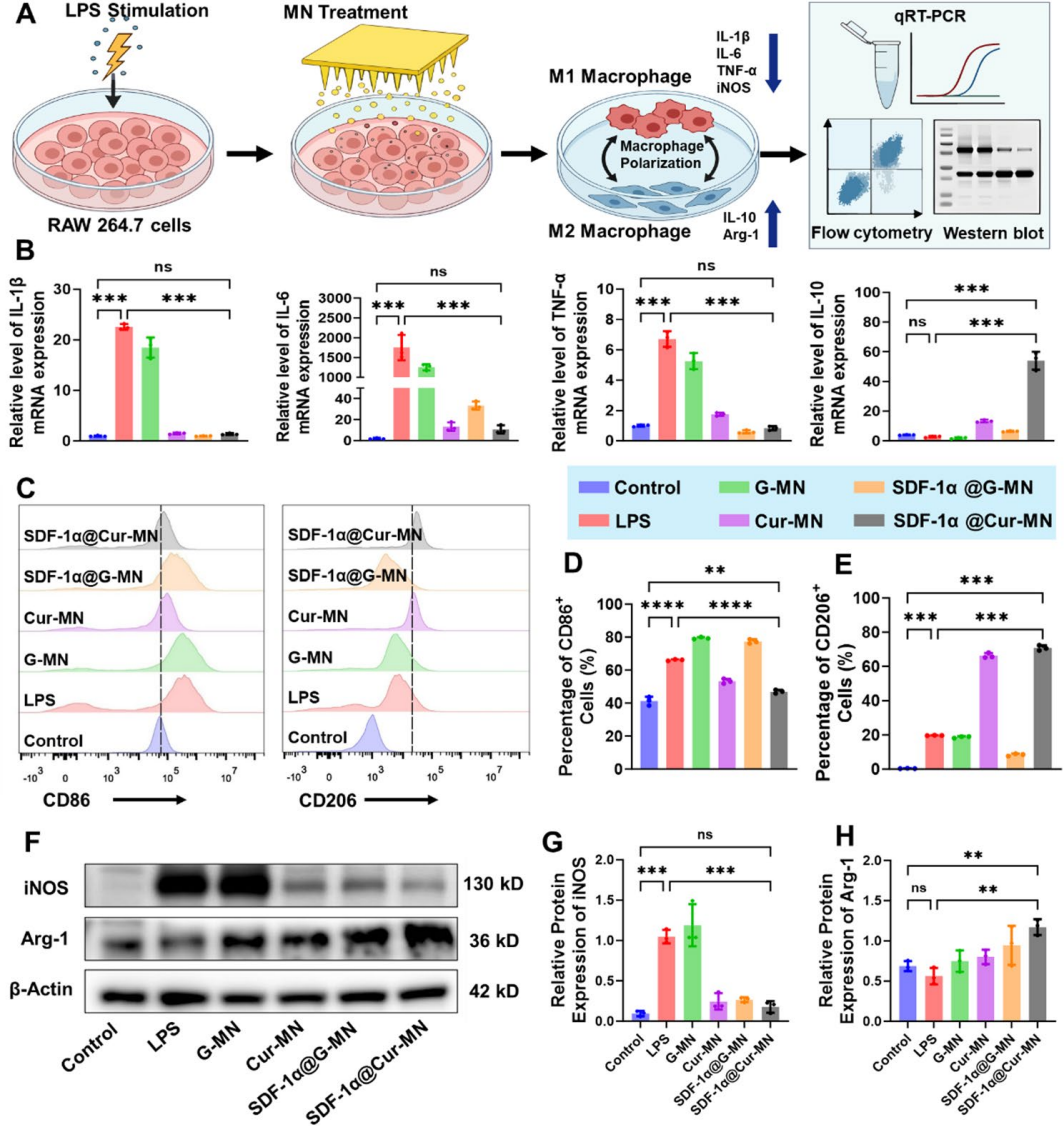
Effect of SDF-1α@Cur-MN on macrophage phenotypic regulation. (**A**) Schematic of the in vitro macrophage polarization experiments. **(B)** mRNA expression levels of pro-inflammatory cytokines (IL-1β, IL-6, and TNF-α) and anti-inflammatory cytokine (IL-10) in LPS-treated RAW 264.7 cells after treatment with different MN patches (*n* = 3). **(C)** Flow cytometric analysis for the proportion of M1 phenotype (CD86^+^) and M2 phenotype (CD206^+^) in RAW 264.7 cells after different treatments. **(D-E)** Quantitative analysis of proportion of M1 phenotype (CD86^+^) and M2 phenotype (CD206^+^) in RAW 264.7 cells after different treatments (*n* = 3). **(F-H)** Western blotting and quantitative analysis of iNOS and Arg-1 expression levels in RAW 264.7 cells after different treatments (*n* = 3). (Quantitative data were presented as mean ± SEM. Statistical significance was assessed using one-way ANOVA, followed by Tukey’s multiple comparisons test. Significant differences were denoted as *p < 0.05, **p < 0.01, ***p < 0.001, and ****p < 0.0001, while ns indicated non-significance.)

The early inflammatory response following MI is a critical process that exacerbates initial injury while setting the stage for subsequent repair [53]. Ischemic cardiomyocytes release damage-associated molecular patterns (DAMPs) (e.g., HMGB1, ATP), which activate resident macrophages and recruit circulating neutrophils and monocytes to the injury site. These macrophages polarize into distinct phenotypes: pro-inflammatory M1 (secreting TNF-α, IL-6) and pro-reparative M2 (characterized by Arg-1 and IL-10). A timely transition from M1 to M2 is essential for inflammation resolution and tissue repair, whereas sustained M1 activation exacerbates injury and adverse remodeling, and early M2 polarization facilitates infarct stabilization and recovery [54]. The effect of SDF-1α@Cur-MN patches on anti-inflammatory and macrophages reprogramming was investigated using qPCR, flow cytometry analysis and Western blotting (Fig. 2A). For comparative purposes, other MN patches including GelMA MN (G-MN), Cur-GelMA MN (Cur-MN), and SDF-1α-loaded GelMA MN (SDF-1α@G-MN) were used as controls.

To evaluate the anti-inflammatory efficacy of the fabricated SDF-1α@Cur-MN, RAW 264.7 macrophages were first stimulated with LPS to establish an inflammatory model (Figure S7), followed by treatment with different MN patches. Unstimulated cells served as the control. As shown in Fig. 2B, LPS treatment significantly induced the pro-inflammatory gene expression, including IL-1β, IL-6, and TNF-α, which are known to propagate local or systemic inflammatory responses and contribute to cardiac injury. Gene expression analysis by RT-qPCR revealed that Cur-MN and SDF-1α@Cur-MN treatment markedly downregulated the expression of these pro-inflammatory genes, while upregulating anti-inflammatory gene IL-10 compared to the LPS-induced group. In parallel, flow cytometry was employed to assess macrophage polarization by detecting the surface markers CD86 (M1) and CD206 (M2) (Fig. 2C-E). The results indicated that both Cur-MN and SDF-1α@Cur-MN reduced the proportion of CD86⁺-M1 macrophages to 53.2% and 46.9%, respectively, relative to the LPS group (66.1%) (Fig. 2D). Conversely, Cur-MN and SDF-1α@Cur-MN treatment promoted a substantially higher percentage of CD206⁺-M2 macrophages (66.3% and 70.8%, respectively) than LPS group (19.6%) (Fig. 2E). Correspondingly, the M1:M2 ratio shifted from 3.4:1 to 0.7:1, highlighting curcumin-mediated polarization toward the regenerative phenotype. Western blotting further validated macrophage phenotypic modulation at the protein level (Fig. 2F-H), showing decreased iNOS and increased Arg-1 protein expression in the Cur-MN and SDF-1α@Cur-MN-treated macrophages. Collectively, qPCR, flow cytometry, and Western blot results converge to demonstrate that SDF-1α@Cur-MN effectively attenuates LPS-induced pro-inflammatory activation and promotes a shift toward a reparative macrophage phenotype. This dual-action modulation, rapid suppression of M1-driven cytokine storm coupled with enhancement of M2 reparative signaling, disrupts the self-perpetuating cycle of inflammation and establishes a favorable microenvironment for cardiac regeneration.

### Pro-angiogenic effects of SDF-1α@Cur-MN patch

**Fig. 3:**
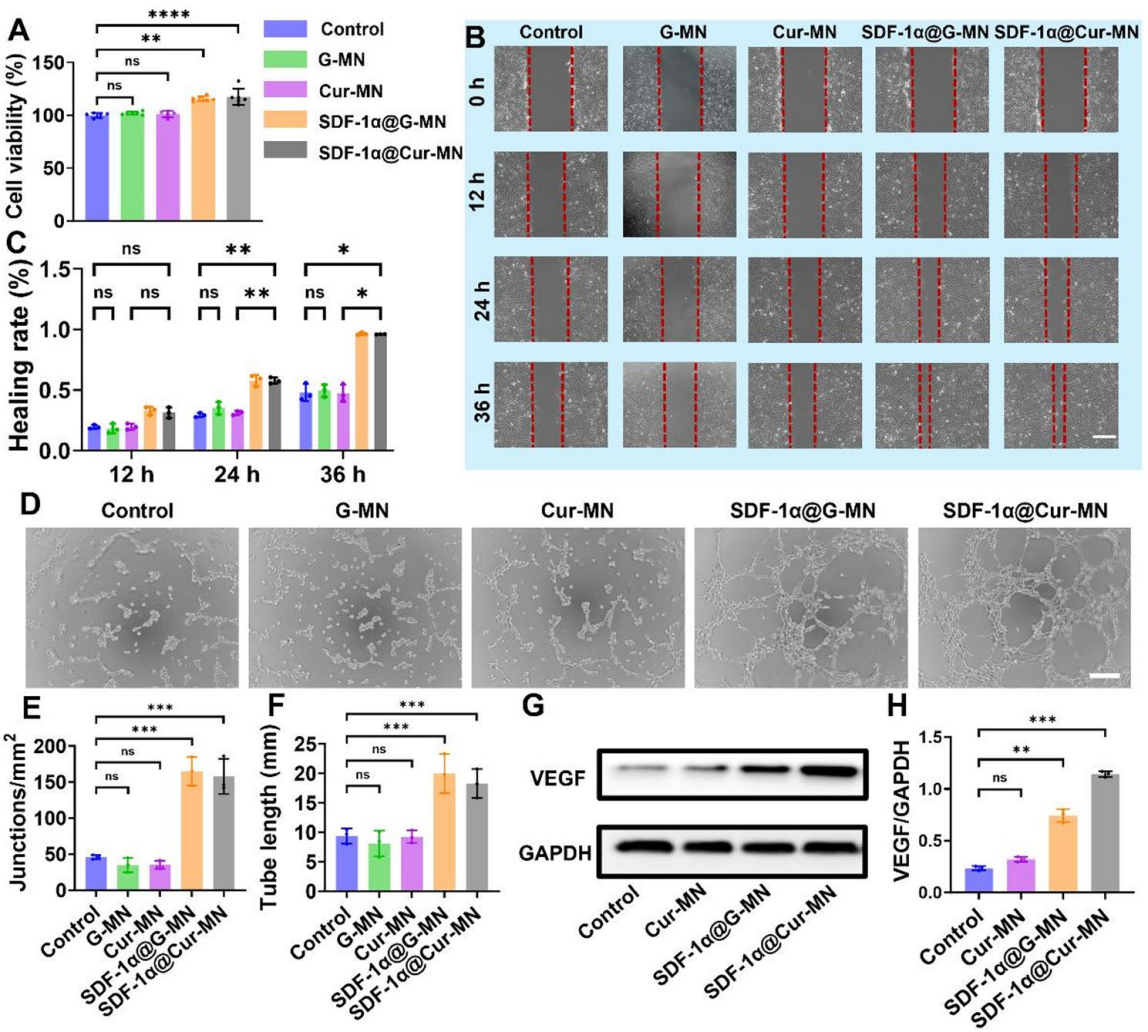
Assessment of the angiogenesis activity of SDF-1α@Cur-MN. **(A)** Cell viability of HUVECs after treatment with different MN patches (*n* = 5). **(B)** Representative bright-field images in migration assays of HUVECs following treatment with different MN patches for intervals of 0, 12 h, 24 h and 36 h. **(C)** Quantitative analysis of migration rates of HUVECs in different groups (*n* = 3). **(D-F)** Representative images of HUVEC tube formation (D) and quantitative analysis of junctions (E) and tube length **(F)** in different groups (*n* = 3). **(G**,** H)** Western blotting **(G)** and quantitative analysis **(H)** of VEGF protein levels in HUVECs following treatment with different MN patches (*n* = 3). (Quantitative data were presented as mean ± SEM. Statistical significance was assessed using one-way ANOVA, followed by Tukey’s multiple comparisons test. Significant differences were denoted as *p < 0.05, **p < 0.01, ***p < 0.001, and ****p < 0.0001, while ns indicated non-significance.)

Angiogenesis is a critical process in myocardial repair following infarction. Based on previous evidence that SDF-1α promotes endothelial cell proliferation and angiogenesis in wound healing contexts, we evaluated the pro-angiogenic potential of SDF-1α@Cur-MN patch using human umbilical vein endothelial cells (HUVECs) [55]. Cell proliferation was assessed using the CCK-8 assay over three days. Both SDF-1α@G-MN and SDF-1α@Cur-MN significantly enhanced HUVEC proliferation compared to the control group (Fig. 3A). By 72 h, cell viability reached 115.8% and 117.5% in the SDF-1α@G-MN and SDF-1α@Cur-MN groups, respectively, markedly higher than G-MN (102.1%) and baseline control (100%) groups, confirming that SDF-1α loading effectively promotes endothelial cell multiplication. We next examined cell migration, a key step in angiogenesis, using a scratch wound assay. As shown in Fig. 3B-C, while minimal migration was observed in the control group, and no significant differences were detected among the control, G-MN, and Cur-MN groups, both SDF-1α-loaded groups significantly enhanced HUVEC migration by 36 h. Migration rates reached 96.4% and 96.0% in the SDF-1α@G-MN and SDF-1α@Cur-MN groups, respectively, substantially higher than in other treatments.

Tube formation assays further demonstrated the angiogenic potential of the formulations. After 6 h of incubation on Matrigel, SDF-1α-loaded groups exhibited the most extensive tube network (Fig. 3D). Quantitative analysis of total tube length and junction numbers confirmed that both SDF-1α@G-MN and SDF-1α@Cur-MN treatment significantly enhanced tube formation compared to other groups (Fig. 3E and F). No significant differences were observed among the Control, G-MN, and Cur-MN groups, indicating that neither GelMA nor Cur-GelMA alone promoted angiogenesis. In contrast, SDF-1α-loaded groups (SDF-1α@G-MN and SDF-1α@Cur-MN) showed more extensive vascular network formation, with statistical analysis of mesh number and total tube length confirming strong stimulatory effect of SDF-1α on HUVEC tube formation. Furthermore, western blot analysis revealed that SDF-1α-loaded MN patches significantly increased VEGF protein expression compared to the normal control (Fig. 3G and H). Taken together, these findings demonstrate that SDF-1α-loaded MN patches effectively promote HUVEC proliferation, migration, and tube formation, likely through upregulation of VEGF expression. These results suggest that SDF-1α delivery via MN patches enhances multiple aspects of angiogenesis, supporting their potential application in promoting myocardial tissue repair after infarction.

### Therapeutic effects of SDF-1α@Cur-MN patch on restoration of cardiac function in a rat MI model

**Fig. 4:**
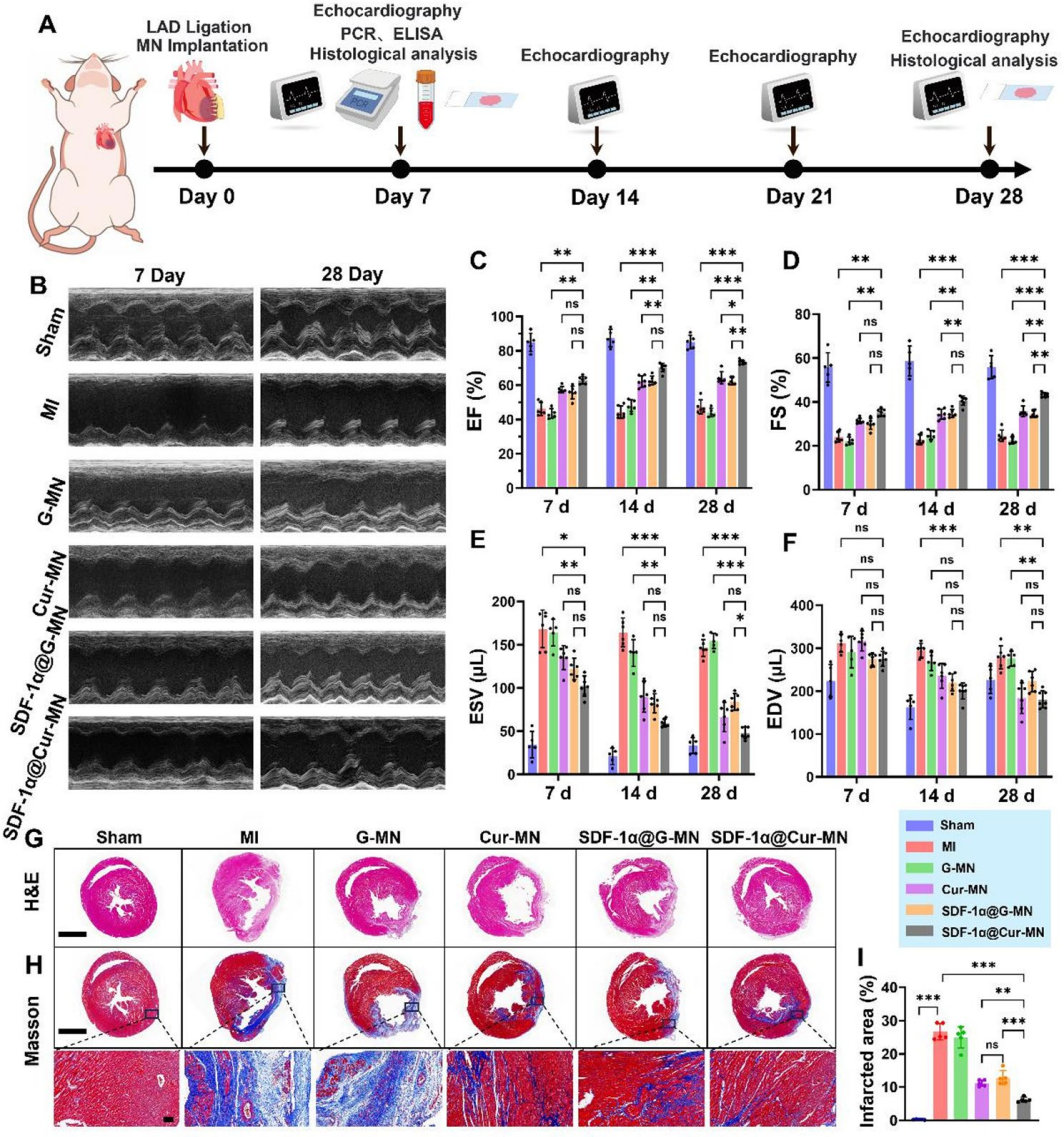
The SDF-1α@Cur-MN patch preserves cardiac function in rats after MI. **(A)** Schematic illustration of the in vivo experimental design for assessing the therapeutic effects of the SDF-1α@Cur-MN patch in a rat MI model. **(B)** Representative M-mode echocardiographic images obtained at 7- and 28-days post-MI. **(C-F)** Echocardiographic assessment of left ventricular function: EF **(C)**, FS **(D)**, ESV **(E)**, and EDV **(F)** following treatment with different MN patches at 7-, 14-, and 28-days post-MI (*n* = 5–8). **(G)** H&E staining of heart tissues at 28 days post-MI. Scale bar: 200 μm. **(H)** Masson’s trichrome staining of heart tissues at 28 days post-treatment. Scale bar: 100 μm. **(I)** Quantitative assessment of infarcted size at 28 days post-MI (*n* = 5–8). (Quantitative data were presented as mean ± SEM. Statistical significance was assessed using one-way ANOVA, followed by Tukey’s multiple comparisons test. Significant differences were denoted as *p < 0.05, **p < 0.01, ***p < 0.001, and ****p < 0.0001, while ns indicated non-significance.)

To evaluate the therapeutic efficacy of SDF-1α@Cur-MN patch on cardiac function, an acute MI model was established by permanent ligation of the left anterior descending (LAD) coronary artery in rats. Immediately following LAD ligation, various MN patches were implanted onto the epicardial surface overlying the infarct zone through the thoracotomy incision. The rats were established as the Sham group with surgery alone, and the other five groups were established with MI and implantation of different MN patches including GelMA MN (G-MN), Cur-GelMA MN (Cur-MN), GelMA MN loaded with SDF-1α (SDF-1α@G-MN), and Cur-GelMA MN loaded with SDF-1α (SDF-1α@Cur-MN). The schedule for echocardiographic assessments and histopathological analyses over 28 days is outlined in Fig. 4A. Cardiac function was assessed by echocardiography at 7-, 14-, and 28-days post-MI treatment (Fig. 4B and Figure S8). Representative M-mode images demonstrated markedly reduced or absent wall motion in the MI group at Day 7, indicating significant systolic dysfunction. In contrast, rats treated with SDF-1α@Cur-MN exhibited progressive recovery of ventricular wall motion throughout the treatment period, with the greatest improvement among all MN-treated groups.

Quantitative analysis of left ventricular performance was conducted using left ventricular ejection fraction (EF) and fractional shortening (FS). As shown in Fig. 4C and D, both EF and FS progressively declined in the MI and G-MN groups over 28 days. Treatment with SDF-1α@Cur-MN significantly preserved systolic function, yielding higher EF (73.51%) and FS (43.21%) at Day 28 compared with MI (EF:47.27%; FS: 24.56%) and other control MN groups, with values approaching the levels observed in sham-operated rats. The single-component-loaded MN patches treatment (Cur-MN and SDF-1α@G-MN groups) exhibited intermediate improvements, though to a lesser degree than the dual-loaded SDF-1α@Cur-MN group. Ventricular remodeling was further assessed by measuring end-diastolic volume (EDV) and end-systolic volume (ESV). As illustrated in Fig. 4E and F, the MI group showed significant increases in both EDV and ESV from Day 7 to Day 28 compared to the Sham group, indicating progressive ventricular dilation. Cur-MN, SDF-1α@G-MN and SDF-1α@Cur-MN treated groups demonstrated attenuated ventricular expansion compared to the MI and G-MN groups, with the SDF-1α@Cur-MN group showing the smallest increases in chamber volumes. Together, quantitative analysis of these functional parameters confirms that dual-loaded SDF-1α@Cur-MN treatment yielded superior preservation of cardiac function compared to Cur-MN or SDF-1α@G-MN alone, suggesting a synergistic therapeutic efficacy between Cur and SDF-1α in promoting myocardial repair.

To further evaluate myocardial injury and ventricular remodeling, hearts were harvested at Day 28 for histological analysis with hematoxylin and eosin (H&E) and Masson’s trichrome staining. H&E staining revealed normal, orderly myofiber alignment without evident necrosis or significant interstitial inflammation in the Sham group, whereas MI and G-MN groups displayed substantial myocyte loss, disarray, and inflammatory infiltration (Fig. 4G). The SDF-1α@Cur-MN groups, however, showed moderately preserved tissue organization with only mild-to-moderate edema and limited vascular hyperplasia in focal areas. Masson’s trichrome staining demonstrated extensive collagen deposition and fibrotic replacement in MI and G-MN hearts (Fig. 4H). In contrast, SDF-1α@Cur-MN treatment markedly reduced cardiac fibrosis, preserved viable myocardium, and attenuated adverse remodeling. Quantitative analysis confirmed that the SDF-1α@Cur-MN group had the smallest infarcted size among all treated groups (Fig. 4I). Notably, single-component-loaded MN patches (Cur-MN and SDF-1α@G-MN groups) also reduced fibrosis compared to MI controls, but their effects were substantially weaker than the dual-loaded SDF-1α@Cur-MN group, demonstrating a synergistic therapeutic efficacy. Collectively, these echocardiographic and histological analyses demonstrate that SDF-1α@Cur-MN patches confer significant cardio-protection and promote structural and functional recovery after MI. The resulting improvements including attenuated ventricular remodeling, reduced infarcted size, and decreased fibrosis, collectively underscore the therapeutic potential of combined delivery of Cur and SDF-1α for post-infarction myocardial repair.

### Anti-inflammatory effects of SDF-1α@Cur-MN patch in the early phase of MI

**Fig. 5:**
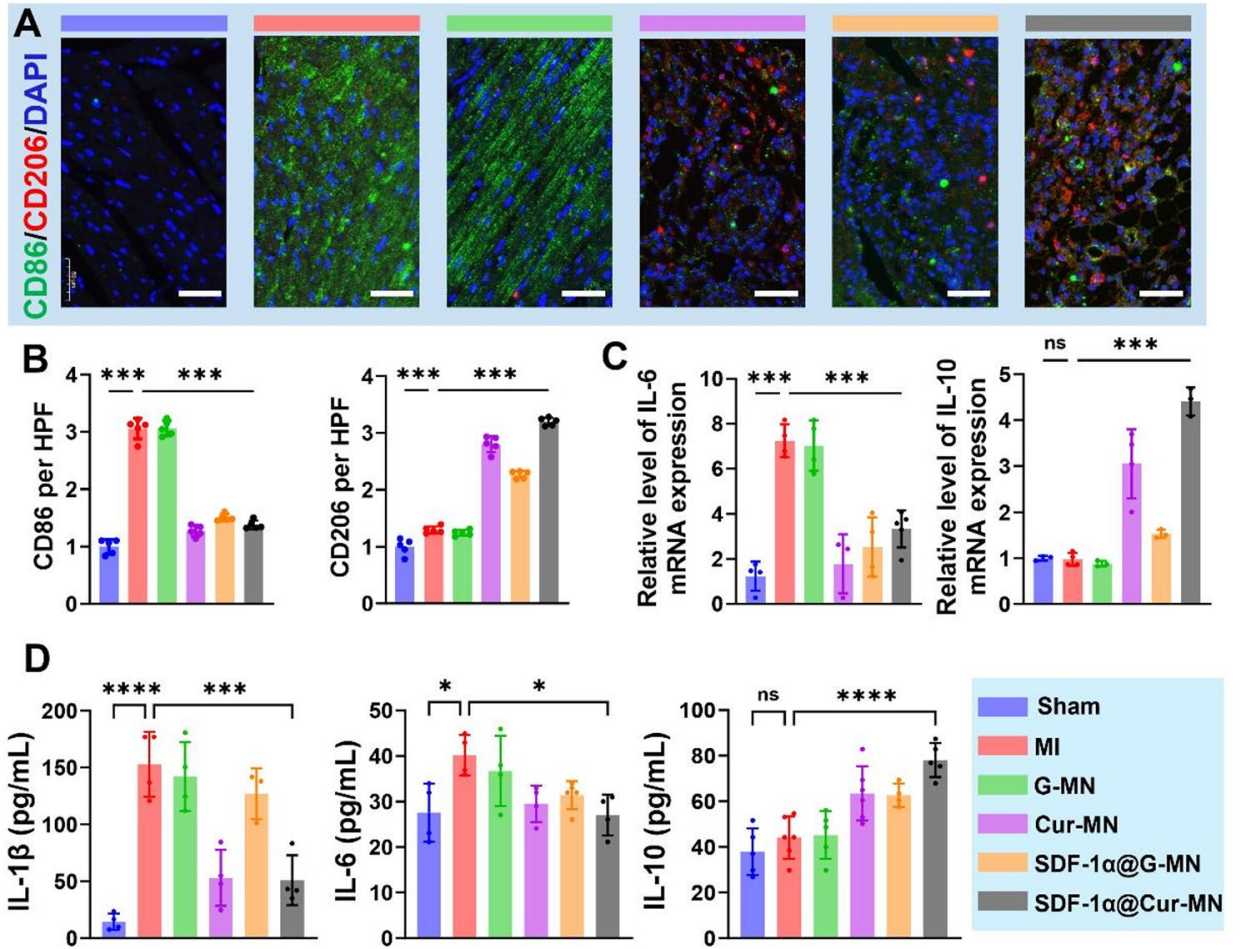
Anti-inflammatory effect of SDF-1α@Cur-MN patch in rat after MI. **(A)** Representative confocal microscopy images of CD86 and CD206 staining of heart sections at 7 days post-MI. Scale bar: 50 μm. **(B)** Quantitative analysis of CD86 and CD206 staining (*n* = 5). **(C)** mRNA expression levels of IL-6 and IL-10 in infarcted heart samples at 7 days post-MI (*n* = 4). **(D)** ELISA results of IL-1β, IL-6, and IL-10 levels in serum at 7 days post-MI (*n* = 4). (Quantitative data were presented as mean ± SEM. Statistical significance was assessed using one-way ANOVA, followed by Tukey’s multiple comparisons test. Significant differences were denoted as *p < 0.05, **p < 0.01, ***p < 0.001, and ****p < 0.0001, while ns indicated non-significance.)

Myocardial infarction triggers a pronounced inflammatory response which activates resident macrophages and recruits circulating neutrophils and monocytes to the ischemic region. Given the pivotal role of inflammation in the early phase of MI, we evaluated the immunomodulatory effects of various MN patches by analyzing cardiac tissues and serum samples collected 7 days post-MI. To assess macrophages polarization in the infarcted myocardium, immunofluorescence staining was performed using CD86 and CD206 as markers for M1 and M2 phenotypes, respectively. As shown in Fig. 5A, in the sham group, minimal macrophage infiltration was observed. In contrast, the MI group exhibited a significant increase in M1 macrophages, indicating a dominant pro-inflammatory state. Cur-loaded MN patches treatments reduced the M1/M2 ratio compared to the MI group, with the most pronounced shift toward the M2 phenotype observed in SDF-1α@Cur-MN group (Fig. 5B). These results suggest that Cur-grafted hydrogel exerts potent anti-inflammatory effects within the infarct microenvironment by promoting the transition from pro-inflammatory M1 to reparative M2 macrophages.

We further analyzed the inflammatory status at the transcriptional level using RT-qPCR on infarct zone tissues. The MI group showed significantly elevated mRNA expression of the pro-inflammatory cytokine IL-6 compared to sham control (Fig. 5C). SDF-1α@Cur-MN groups downregulated IL-6 expression. Concurrently, this group also exhibited the highest upregulation of the anti-inflammatory cytokine IL-10, indicating a robust shift toward an inflammation-resolving microenvironment. Consistent with these findings, ELISA of serum samples collected on day 7 post-MI revealed markedly elevated levels of IL-1β and IL-6 in the MI group (Fig. 5D). Treatment with SDF-1α@Cur-MN significantly reduced the concentrations of these pro-inflammatory cytokines while enhancing IL-10 level, further confirming its systemic anti-inflammatory efficacy. These results demonstrate that SDF-1α@Cur-MN patch effectively modulate post-infarction inflammation by skewing macrophage polarization toward the M2 phenotype and rebalancing the expression of key inflammatory mediators. This coordinated anti-inflammatory response provides the therapeutic potential of the dual-loaded MN system in mitigating early inflammatory damage and creating a conducive microenvironment for myocardial repair.

### Evaluation of anti-inflammatory, anti-apoptotic, and pro-angiogenic effects of SDF-1α@Cur-MN patch post-MI

**Fig. 6:**
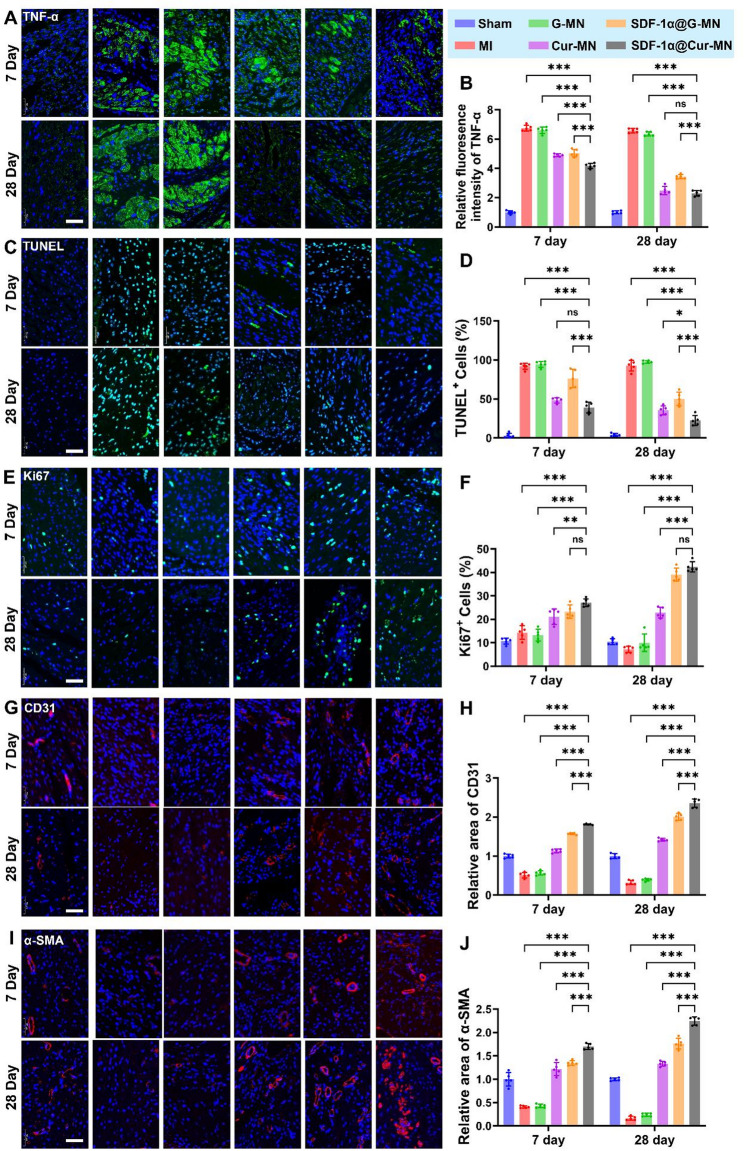
Confocal microscopic analysis of cardiac sections at 7- and 28-days post-MI. **(A**,** B)** IF staining images (**A**) and quantified data (**B**) of TNF-α. **(C**,** D)** TUNEL staining images (**C**) and quantified data (**D**) of apoptotic cells. **(E**,** F)** IF staining images (**E**) and quantified data (**F**) of Ki67. **(G**,** H)** IF staining images (**G**) and quantified data (**H**) of CD31 (angiogenesis marker). **(I**,** J)** IF staining images (**I**) and quantified data (**J**) of α-SMA (smooth muscle actin, vessel maturation marker). Scale bars: 50 μm. (Quantitative data were presented as mean ± SEM. Statistical significance was assessed using one-way ANOVA, followed by Tukey’s multiple comparisons test. Significant differences were denoted as *p < 0.05, **p < 0.01, ***p < 0.001, and ****p < 0.0001, while ns indicated non-significance.)

To assess inflammation, apoptosis, proliferation, and angiogenesis in vivo, myocardial tissues from the infarcted region harvested on day 7 and 28 post-MI were examined by immunofluorescence staining. First, immunofluorescence staining for TNF-α, a key proinflammatory mediator, revealed significantly elevated expression in the MI group compared to Sham group at both 7- and 28-days post-MI, with no notable difference between the MI and G-MN groups (Fig. 6A). Both single-loaded and dual-loaded MN patches treatments markedly suppressed TNF-α expression, with the dual-drug MN group (SDF-1α@Cur-MN) exhibiting the strongest inhibition (Fig. 6B). By day 28, TNF-α expression was progressively decreased in the Cur-MN and SDF-1α@Cur-MN groups, confirming the in vivo anti-inflammatory activity of Cur-grafted hydrogels. TUNEL staining revealed extensive cellular apoptosis in the MI group, reaching 92.87% at day 28 (Fig. 6C and D). In contrast, Cur-MN and SDF-1α@Cur-MN patches significantly reduced apoptosis to 35.53% and 22.38%, respectively, indicating Cur’s efficacy in mitigating cell death within the ROS-enriched MI microenvironment. Further TUNEL evaluation at days 7 and 28 confirmed substantially increased apoptosis in the MI group relative to Sham control (Fig. 6C). Notably, apoptosis in the SDF-1α@Cur-MN group further decreased from day 7 to day 28, reflecting sustained therapeutic benefit.

Cardiomyocyte proliferation, a critical step in post-MI repair, was assessed by Ki67 staining. The SDF-1α@G-MN and SDF-1α@Cur-MN groups exhibited significantly enhanced cellular proliferation compared to the Sham, MI, and G-MN groups at both time points (Fig. 6E). The SDF-1α@Cur-MN group showed the strongest proliferative response, with the highest absolute levels and most rapid proliferation rate among all treatments (Fig. 6F). Angiogenesis was evaluated through CD31 and α-SMA immunostaining (Fig. 6G-J). On day 7, CD31 and α-SMA expression was significantly impaired in the MI and G-MN groups compared to Sham, whereas the SDF-1α-loaded MN groups (SDF-1α@G-MN and SDF-1α@Cur-MN) maintained higher CD31 levels comparable to Sham and exhibited significantly higher α-SMA expression than the MI and Cur-MN groups. By day 28, CD31 and α-SMA expression was nearly absent in MI and MN groups, whereas all treatment groups, particularly SDF-1α@Cur-MN, showed markedly elevated expression of both markers, suggesting the formation of mature and functional vasculature. These analyses demonstrate that dual-target therapy exert comprehensive cardioprotective effects through coordinated attenuation of apoptosis and inflammation while promoting angiogenesis and cellular proliferation.

## Discussion

Myocardial infarction (MI) initiates a complex pathological cascade involving excessive inflammation, massive cardiomyocytes death, and inadequate angiogenesis, collectively driving adverse ventricular remodeling and heart failure. Despite extensive development of therapeutic strategies targeting individual pathological processes, their clinical efficacy remains limited by the inability to spatiotemporally coordinate multiple repair mechanisms within the dynamic infarct microenvironment [3–5]. In this study, we developed a curcumin hydrogel-based microneedle patch encapsulating SDF-1α (SDF-1α@Cur-MN) capable of co-delivering curcumin and SDF-1α in a temporally-delivered manner. This system couples early anti-inflammatory intervention with sustained pro-angiogenic activation for promoting cardiac repair.

One of the key innovations of this work lies in the rational design of the Cur-GelMA hydrogel. By conjugating curcumin onto GelMA via phenylboronic acid (PBA) ester bonds, we achieved a significant enhancement in curcumin solubility and bioavailability, as well as conferred ROS-responsive release behavior. This chemical design effectively addresses the major pharmacokinetic limitations of curcumin, including extreme hydrophobicity and rapid metabolism, while ensuring targeted drug availability during the early inflammatory phase.

The subsequent integration of the hydrogel into MN patches via PDMS micromolding yielded a robust epicardial patch with excellent tissue penetration capability and mechanical stability. This MN platform ensures targeted penetration into the infarcted region, enabling localized high-concentration delivery while minimizing systemic exposure and overcoming conventional targeting limitations. The homogeneous distribution of therapeutics throughout both the microneedle tips and the backing layer was chosen for the design of MN patch. Unlike conventional transdermal MN delivery where only the tips are drug-loaded, our cardiac application requires a single-dose, surgically implanted device capable of delivering sufficient payload to support sustained release over weeks. Accordingly, the backing layer serves as a drug reservoir, substantially increasing total drug loading. Upon implantation, the microneedle tips penetrate the myocardial tissue and provide two critical functions. First, they confer mechanical anchorage, as evidenced by a 4.5-fold increase in adhesion strength compared to bulk patches (~ 14 kPa vs. ~3 kPa, Figs. 1J-K), ensuring reliable retention on the beating heart. Second, they create microchannels that facilitate direct drug diffusion into deep myocardial layers. Simultaneously, the backing layer maintains intimate contact with the epicardial surface, enabling continuous drug diffusion across the tissue interface. This dual delivery pathway, combined with the differential release kinetics of covalently conjugated curcumin (ROS-responsive) and physically encapsulated SDF-1α (sustained), enables spatiotemporally coordinated therapy with a single administration.

Our in vitro and in vivo results robustly demonstrate the dual-pathway efficacy of the SDF-1α@Cur-MN patch. A pertinent consideration is the potential anti-angiogenic effect of curcumin reported in cancer models. However, curcumin’s effects are highly context-dependent. In myocardial infarction, curcumin promotes a pro-reparative rather than anti-angiogenic role by resolving inflammation and facilitating M2 polarization, thereby creating a permissive microenvironment for subsequent vascularization [56]. Our results confirm that curcumin hydrogel significantly skewed macrophage polarization from a pro-inflammatory M1 phenotype toward a reparative M2 state, as evidenced by the downregulation of IL-1β, TNF-α, IL-6, and iNOS and the upregulation of Arg-1 and IL-10. Importantly, Cur-MN treatment alone didn’t impair endothelial cell function in vitro. The temporal coordination in our system ensures rapid curcumin release within 48 h to target early inflammation (Fig. 1L), while sustained SDF-1α release drives later angiogenesis (Fig. 1M). This sequential delivery ensures that curcumin’s anti-inflammatory action precedes and facilitates SDF-1α-driven vascularization, explaining the synergistic outcomes observed. This immunomodulatory effect is particularly crucial since sustained M1 macrophage activation is known to propagate inflammatory damage and impede tissue repair [7, 8, 10]. The observed shift in the M1/M2 ratio and the consequent mitigation of apoptosis in the infarct border zone emphasize curcumin’s role in stabilizing the early MI microenvironment. These findings align with emerging literatures highlighting macrophage reprogramming in ischemic heart disease [11, 57, 58].

Concurrently, the sustained release profile of SDF-1α from the same MN patch ensured continuous angiogenesis during the proliferative phase of cardiac repair. The robust pro-angiogenic effects were manifested through enhanced endothelial cell proliferation, migration, and tube formation in vitro, coupled with significantly increased densities of CD31⁺ and α-SMA⁺ vessels in vivo. The functional benefits of this coordinated MN patches were clearly demonstrated in a rat MI model, where the SDF-1α@Cur-MN treatment yielded substantially better effect than single-component therapies. The significant improvements in left ventricular function, coupled with reduced fibrosis and enhanced tissue preservation, highlight the advantage of simultaneously targeting multiple pathological pathways. More importantly, the temporal coordination between curcumin-driven inflammation resolution and SDF-1α-mediated angiogenesis appears to generate synergistic effects. This aligns with recent studies emphasizing the crucial cross-talk between inflammatory and angiogenic pathways in cardiac regeneration [59, 60], where the anti-inflammatory action of curcumin likely creates a more permissive microenvironment for SDF-1α-driven vascularization. Our approach, which integrates nanoscale material design through stimulus-responsive drug-polymer conjugates with a microscale delivery device, addresses the macroscopic challenge of cardiac repair and embodies the multiscale integration central to advanced nanobiotechnology.

Despite these promising results, several limitations should be acknowledged. While the Franz diffusion cell provided valuable comparative release kinetics, it couldn’t fully replicate the complex in vivo microenvironment. Factors including limited fluid volume, enzymatic degradation, and dynamic tissue motion may modulate actual release rates. Therefore, these data should be interpreted as comparative kinetic characterization rather than precise quantitative predictions. Future studies employing ex vivo perfusion models or in vivo microdialysis techniques could provide more direct quantitative insights into the actual release dynamics within the myocardial tissue. Additionally, the long-term stability, biodegradability, and potential immune response to the implanted patch require further investigation in large-animal models. Future work should focus on scaling up the fabrication process, evaluating biocompatibility and safety in higher species, and exploring the incorporation of other therapeutic agents (e.g., miRNAs or exosomes) to further enhance regenerative outcomes [61, 62]. Personalized approaches, such as tailoring patch dimensions or drug ratios based on infarct characteristics, could also be explored.

## Conclusion

In conclusion, we have developed a curcumin hydrogel-based microneedle patch encapsulating SDF-1α (SDF-1α@Cur-MN) that coordinates anti-inflammatory and pro-angiogenic functions through spatiotemporally controlled release. The Cur-GelMA hydrogel network not only significantly enhanced curcumin’s loading efficiency and bioavailability, but also enabled ROS-responsive release, allowing rapid anti-inflammatory action in the early phase of MI. Combined with the sustained release of SDF-1α, this system effectively coordinated early inflammation suppression with prolonged pro-angiogenic stimulation. In vitro and in vivo results demonstrated that SDF-1α@Cur-MN patch promoted macrophage polarization toward the reparative M2 phenotype, reduced cardiomyocyte apoptosis, enhanced angiogenesis, and improved recovery of cardiac function. The synergistic efficacy of the dual-drug system was clearly superior to single-component treatments, highlighting the therapeutic advantage of simultaneously targeting multiple pathological processes. This work provides a potential strategy for comprehensive myocardial repair through an integrated biomaterial-based delivery platform.

## Electronic Supplementary Material

Below is the link to the electronic supplementary material.

Supplementary Material 1.

## Data Availability

No datasets were generated or analysed during the current study.
