# Supplementary material for: A spatiotemporally coordinated curcumin-based microneedle patch for SDF-1α delivery and synergistic myocardial infarction therapy
Source: J Nanobiotechnology. 2026 Apr 26;24:564. doi: 10.1186/s12951-026-04473-4 (PMC13273951; doi:10.1186/s12951-026-04473-4)
Supplement: Supplementary file 1 — Supplementary Material 1. [file 12951_2026_4473_MOESM1_ESM.doc]

**Supporting information for**

**A** **Spatiotemporally Coordinated Curcumin-Based Microneedle Patch for SDF-1α Delivery and Synergistic Myocardial Infarction Therapy**

Xue-Yan Jiang^1,#^, Yuan Luo^1,#^, Yang Yang^1,#^, Da-Wei Tang^1^, Zhizhong Wang^1^, Pei Huang^1^, Fang-Zhen Wang^1^, Shu-Meng Zhang^1^, Hao-Min Zhang^1^, Yi-Yun Ma^1^, Xu-Chen Liu^1^, Yun-Ru Li^1^, Wenhua Zheng^2^, Lingmin Zhang^1^,*, Xi-Yong Yu^1^,*, Gen He^1^,*

^1^Key Laboratory of Molecular Target & Clinical Pharmacology, the NMPA and State Key Laboratory of Respiratory Disease, School of Pharmaceutical Sciences, Guangzhou Medical University, Guangzhou, 511436, China.

^2^Department of Pharmaceutical Science, Faculty of Health Sciences, University of Macau, Taipa, 999078, Macau, China

**^#^The first authors contributed equally to this work.**

***Corresponding authors:** zhanglm@gzhmu.edu.cn (Ling-min Zhang); [yuxycn@gzhmu.edu.cn](mailto:yuxycn@gzhmu.edu.cn) (Xi-Yong Yu); hegen@gzhmu.edu.cn (Gen He)

**This supplemental material includes Figure S1-S8.**

**
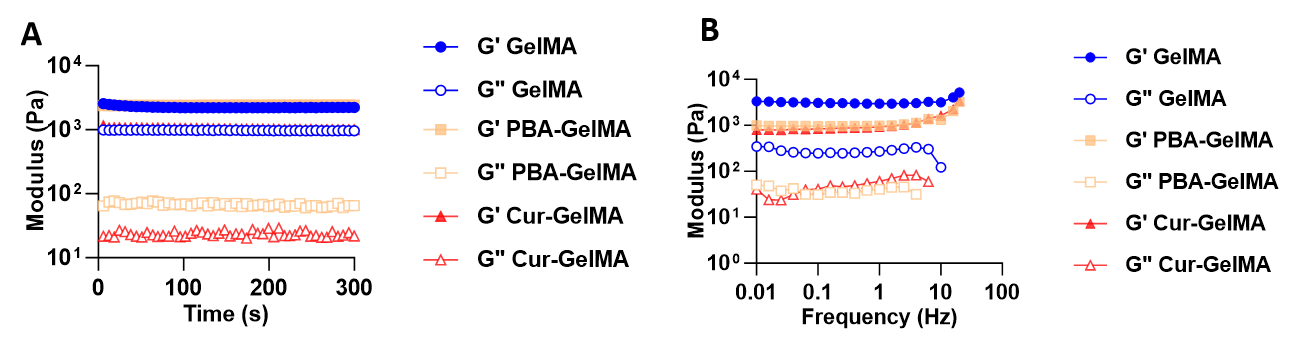
**

**Figure S1. Rheological characterization of hydrogels.** (A) Representative time sweep curves showing the evolution of storage modulus (G′) and loss modulus (G″) during photo-crosslinking of GelMA, PBA-GelMA, and Cur-GelMA. (B) Frequency sweep curves of the three hydrogel formulations measured at 37 °C, demonstrating gel-like behavior (G > G″) across the entire frequency range.


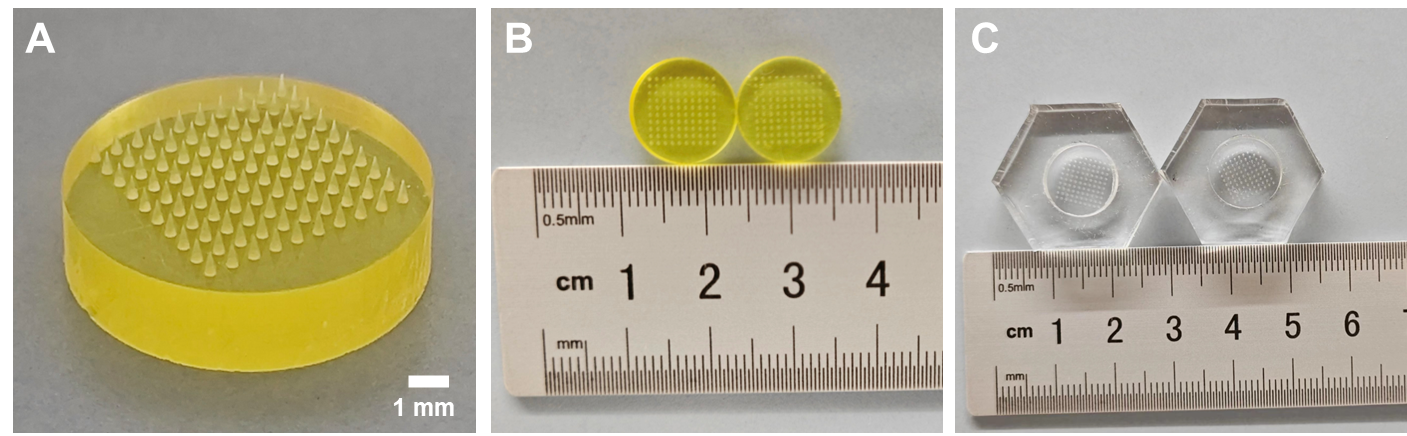


**Figure S2.** Optical images of 3D-printed resin mold **(A**, **B)**, and PDMS-based microneedle molds **(C)**.


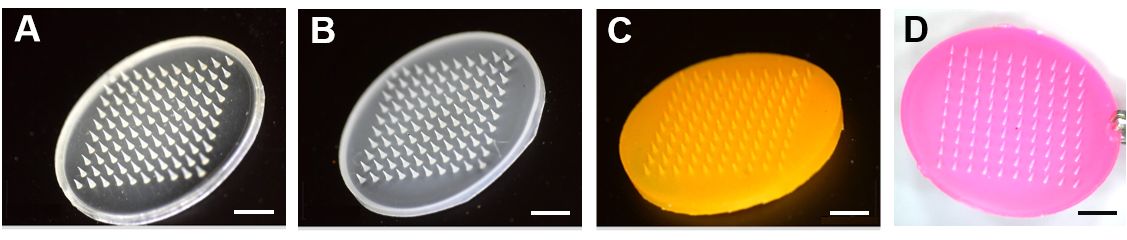


**Figure S3.** Photographs of microneedle patches made of different hydrogels. GelMA-MN **(A)**, PBA-GelMA-MN **(B)** and Cur-MN **(C)**. (**D**) Fluorescence microscopy image of a rhodamine B (RhB)-loaded Cur-MN patch, showing uniform distribution of RhB (red fluorescence) throughout both the microneedle tips and the backing layer. Scale bars: 2000 μm.


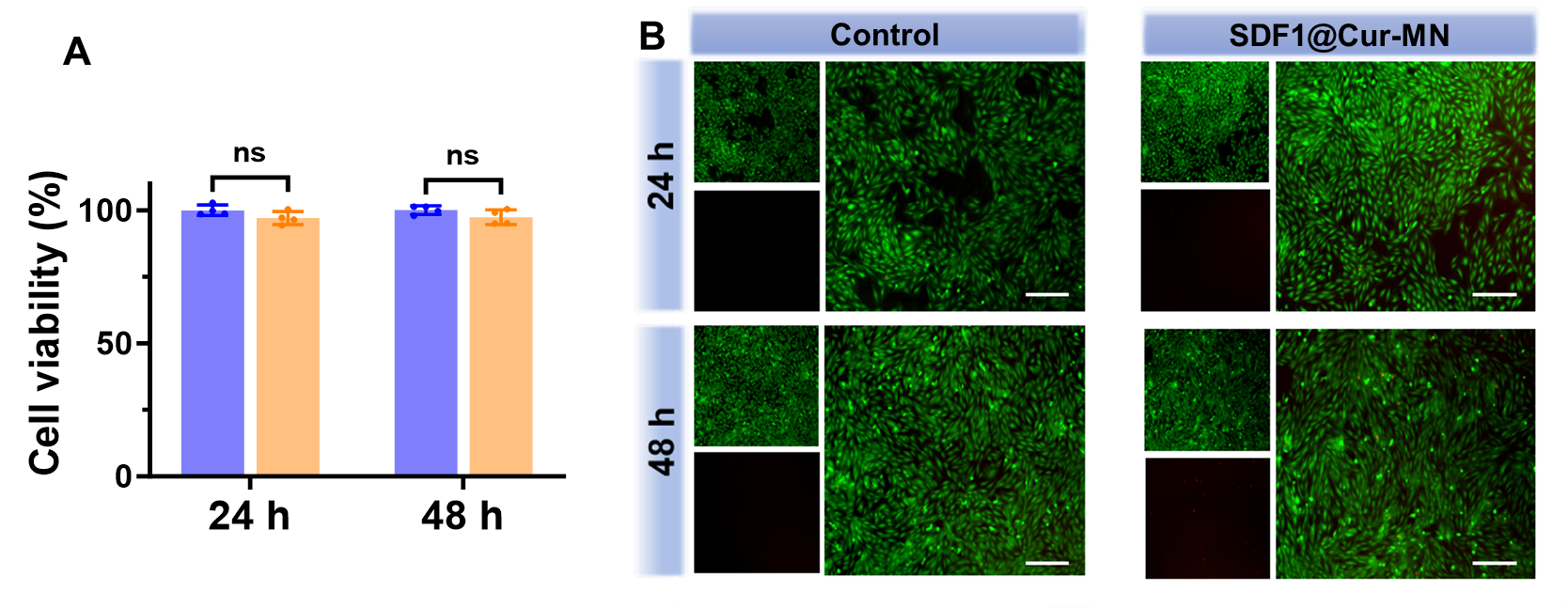


**Figure S4. Biocompatibility of SDF-1α@Cur-MN patches.** **(A)** Cell viability of H9C2 cardiomyocytes treated with SDF-1α@Cur-MN patches for 24 h and 48 h (n=6). **(B)** Fluorescence images of H9C2 cardiomyocytes treated with SDF-1α@Cur-MN patch at day 1, and 2 after double staining with calcein AM and propidium iodide (PI). Scale bar: 200 μm.


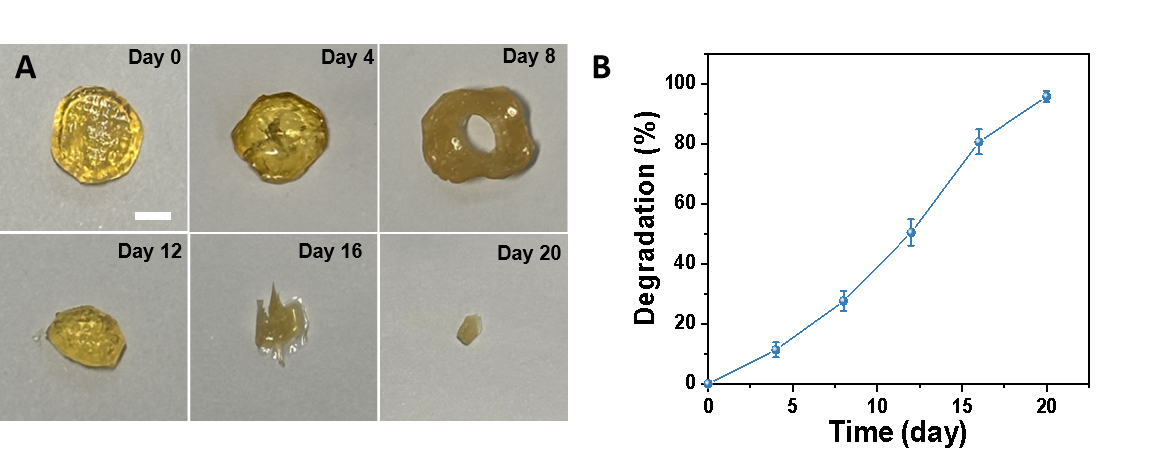


**Figure S5. Time-dependent in vitro degradation behavior of SDF-1α@Cur-MN patches.** **(A)** Representative optical images of SDF-1α@Cur-MN patches after immersed in PBS (pH=7.4, 37 °C) at designated timepoints. **(B)** Quantitative results of time-dependent in vitro degradation rates of SDF-1α@Cur-MN patches. Data are presented as mean ± SEM (n=3).


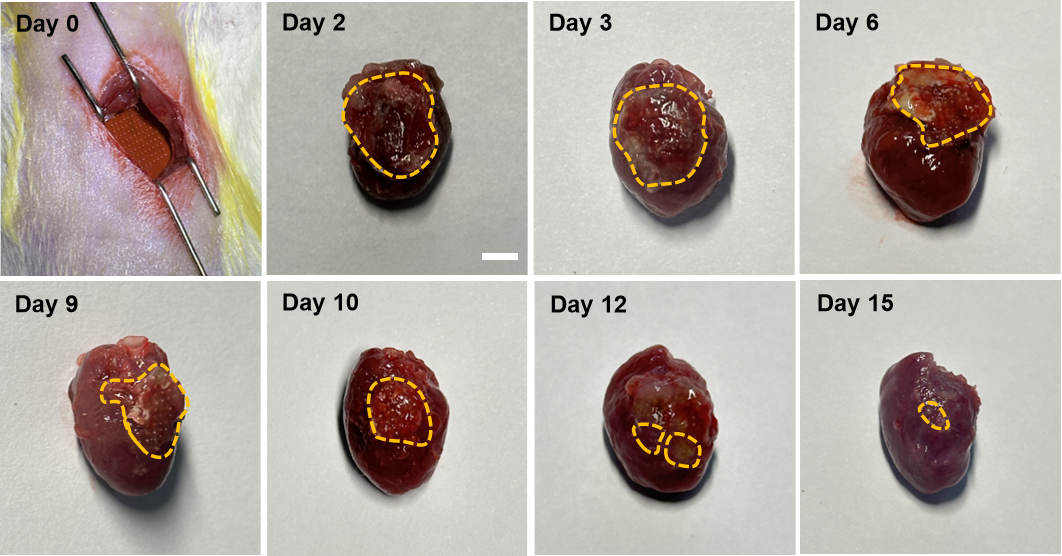


**Figure S6.** **Time-dependent in vivo degradation behavior of SDF-1α@Cur-MN patches after implanted onto the heart in a rat model.** Representative photographs of the rat heart surface at 2-, 3-, 6-, 9-, 10-, 12, and 15-days post-implantation of the SDF-1α@Cur-MN patch, illustrating the progressive in vivo degradation of the hydrogel. The areas marked by the dashed lines are the undegraded MN patches. Scale bar: 1 cm.


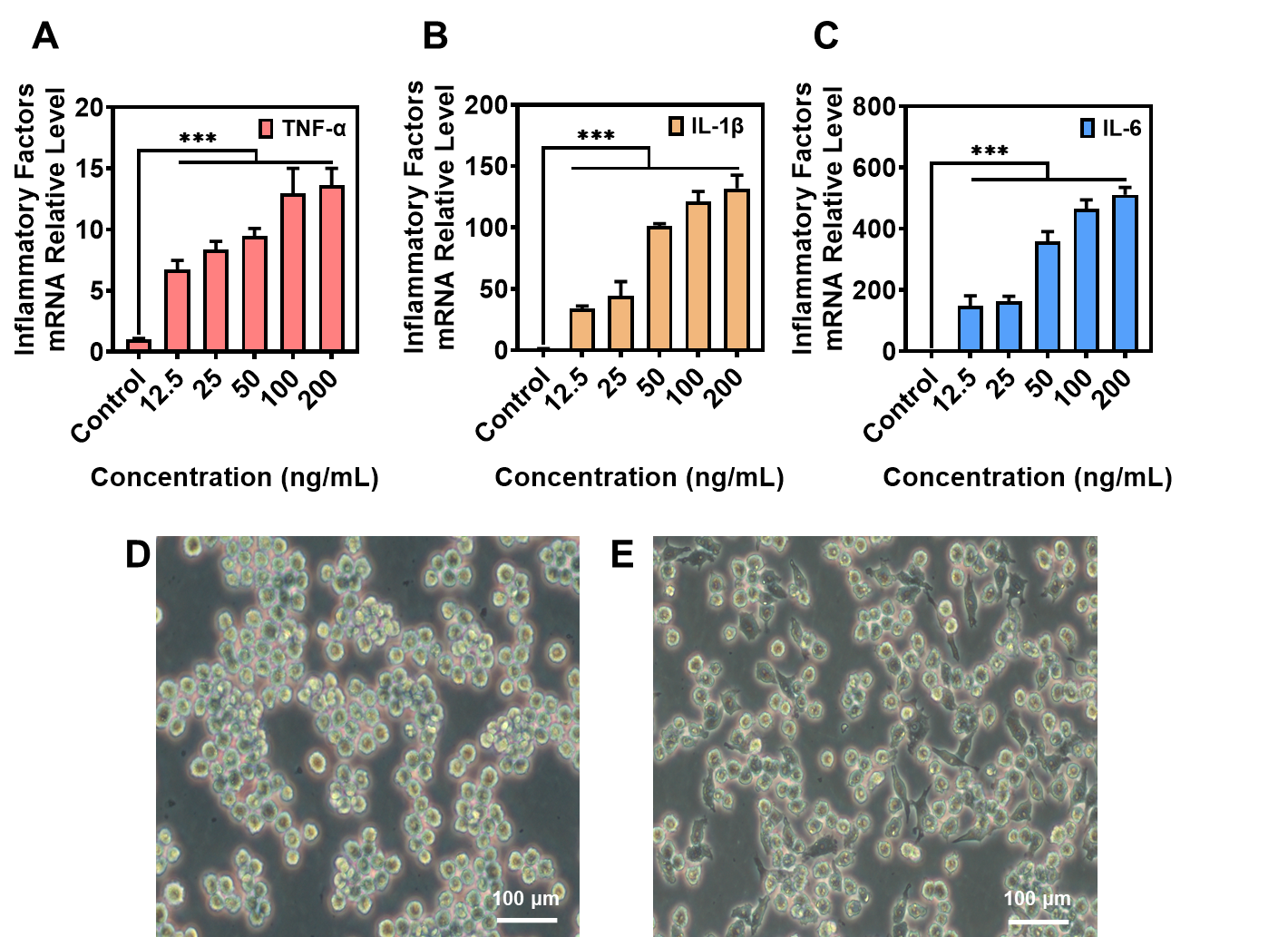


**Figure S7. Different concentrations of LPS stimulated the mRNA expression of inflammatory factors.** **(A-C)** TNF-α, IL-1β, and IL-6 mRNA expression in RAW264.7 after stimulated by different concentrations of LPS (n=4). **(D)** Morphology of RAW264.7 in normal condition. **(E)** Morphology of RAW264.7 after 12 h of LPS stimulation. ***p＜0.001.


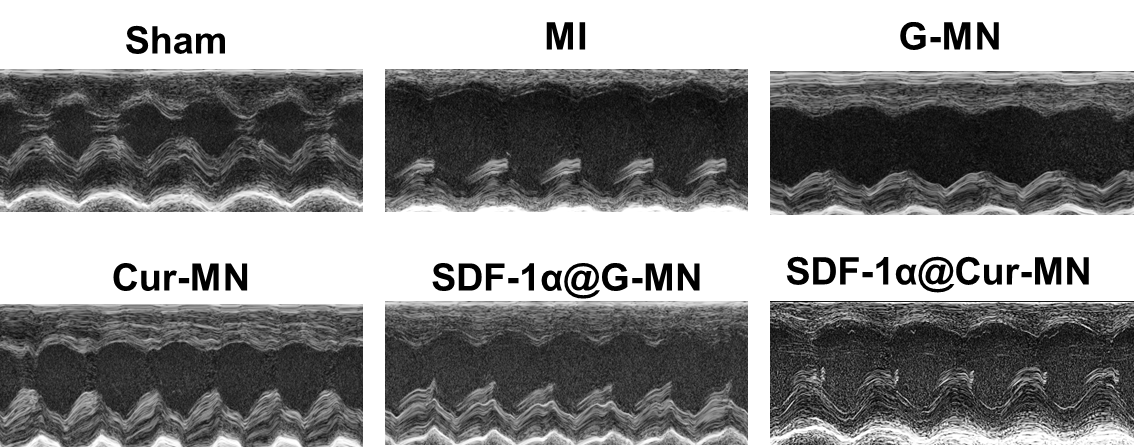


**Figure S8.** Representative M-mode echocardiographic images obtained at 14-days post-MI.
